# Smart Nanoparticles Disrupting Energy Supply through Triple Mechanisms to Kill Tumors via Dual Disruption of Mitochondria and Lysosomes

**DOI:** 10.1002/advs.202517373

**Published:** 2025-11-26

**Authors:** Xiao Xu, Qiqing Huang, Yang Liu, Jinzhuo Liu, Deyi Yang, Yanni Song, Xin Han

**Affiliations:** ^1^ Jiangsu Key Laboratory for Pharmacology and Safety Research of Chinese Materia Medica School of Medicine Nanjing University of Chinese Medicine Nanjing 210023 China; ^2^ Department of Medical Oncology National Cancer Center/National Clinical Research Center for Cancer/Cancer Hospital & Shenzhen Hospital Chinese Academy of Medical Sciences and Peking Union Medical College Shenzhen 518116 China; ^3^ Department of Breast Surgery Harbin Medical University Cancer Hospital 150 Haping Road Harbin 150081 China

**Keywords:** autophagy inhibition, cancer metabolism, lysosomal disruption, macropinocytosis, methuosis, mitochondrial targeting

## Abstract

Targeting mitochondrial disruption as a strategy for inhibiting cancer cell proliferation presents a promising therapeutic approach. However, the process of mitophagy plays a protective role in cancer cells by aiding in damage repair, regulating energy metabolism, and promoting the development of drug resistance. Therefore, designing precise therapies that selectively damage mitochondria while inhibiting mitophagy remains a challenge. This study develops a biomimetic nanoplatform (MTCA@C) with hollow MnO_2_ as the core, loaded with tetrandrine and mitochondrial‐targeted photosensitizer (Ce6‐Apt), and coated with a cell membrane. Under the targeting of the ligand and irradiation of external near‐infrared light, Ce6‐Apt reaches the mitochondria to induce photodynamic reactions causing mitochondrial dysfunction, while also activating mitophagy. Tetrandrine induces lysosomal alkalinization, effectively disrupting the mitophagic flow, and Tet also causes macropinocytosis, characterized by excessive intracellular vacuole accumulation and expansion, leading to cell rupture and ultimately inducing methuosis. Notably, the synergistic effect of these three mechanisms cuts off the energy supply of tumor cells, achieving spatiotemporally controlled precision therapy. In summary, a biomimetic nanoplatform is designed that precisely disrupts the interaction between mitochondria and lysosomes to impair the compensatory energy supply of tumors, addressing the challenge of drug resistance in cancer treatment.

## Graphical abstract

This study introduces a biomimetic nanoplatform (MTCA@C) designed for precise cancer therapy. It targets mitochondria to induce dysfunction through photodynamic activation, while concurrently blocking mitophagy via lysosomal alkalinization. The platform further triggers methuosis, resulting in tumor cell death through vacuolization and energy pathway disruption, offering a spatiotemporally controlled strategy to overcome treatment resistance.

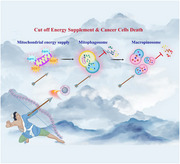

## 1 Introduction

Mitochondria and lysosomes, as two major dynamic organelles within eukaryotic cells, play core roles in energy metabolis^[^ ^1^ ^]^ and material degradation,^[^ ^2^ ^]^ respectively. Their coordinated interaction is crucial for maintaining cellular homeostasis.^[^ ^3^ ^]^ Numerous studies have confirmed that mitochondria and lysosomes communicate physically and functionally.^[^ ^4^, ^5^, ^6^ ^]^ In mammalian cells, lysosomes facilitate contact with mitochondria by regulating the hydrolysis of Rab7, driving mitochondrial fission.^[^ ^7^, ^8^ ^]^ Additionally, mitophagy, which involves lysosomal degradation of damaged mitochondria, is closely related to the progression of clinical diseases.^[^ ^9^, ^10^ ^]^ Mitophagy, or the selective engulfment of damaged mitochondria into autophagosomes followed by lysosomal degradation into nutrients reusable by the cell, is considered a protective mechanism activated when mitochondria are damaged.^[^ ^11^ ^]^ When lysosomes are impaired, mitophagy is obstructed, leading to the accumulation of damaged mitochondria and the production of reactive oxygen species (ROS), exacerbating cellular damage.^[^ ^12^, ^13^ ^]^ Early results from clinical trials using hydroxychloroquine for cancer suggest that inhibiting autophagy may be an effective strategy for treating advanced cancers.^[^ ^14^ ^]^ The interaction between mitochondria and lysosomes thus becomes a key determinant of cellular function and associated diseases. Therefore, developing therapeutic strategies that inhibit the interaction between mitochondria and lysosomes is critical.

Photodynamic therapy (PDT) is a commonly used method to target mitochondria.^[^ ^15^ ^]^ PDT involves a photochemical reaction occurring within cells, with limited diffusion distance, capable of inducing local cell death.^[^ ^16^, ^17^ ^]^ Localizing the photosensitizer within mitochondria could enhance the mitochondrial damage effect of PDT. Aptamers are single‐stranded oligonucleotides characterized by high target specificity and affinity, and they can self‐assemble into complex tertiary and quaternary structures,^[^ ^18^ ^]^ enabling their application in various scenarios. The potential of linking mitochondrial‐targeting aptamers^[^ ^19^ ^]^ with the photosensitizer Ce6 for mitochondrial attack is worth exploring. Damage to mitochondria activates the mitochondrial autophagy pathway, attempting to restore homeostasis within cancer cells, which is also a significant challenge researchers need to address in overcoming tumor drug resistance.

Previous studies have indicated that certain biomaterials can effectively disrupt lysosomes and block autophagy, such as titanium‐coated gold nanoparticles^[^ ^20^, ^21^ ^]^ and black phosphorus nanosheets.^[^ ^22^ ^]^ Additionally, hydroxychloroquine (HCQ) is the most widely used lysosomal alkalinizing agent,^[^ ^23^ ^]^ yet literature indicates that HCQ is limited by its inability to accumulate to sufficient concentrations in many solid tumors to exert its autophagic inhibitory effects.^[^ ^24^ ^]^ Tetrandrine (Tet), a compound extracted from the root of the traditional Chinese medicinal herb Stephania tetrandra S. Clinically, it is primarily used for anti‐rheumatic and analgesic purposes, as well as in the treatment of lung cancer and silicosis.^[^ ^25^, ^26^ ^]^ Existing literature suggests that Tet, a dibenzylisoquinoline derivative, possesses a chemical structure with two nitrogen atoms that enables it to neutralize the acidic environment within lysosomes.^[^ ^27^, ^28^ ^]^ In this study, we selected Tet as the lysosomal alkalinizer for our research by comparing the cytotoxic effects of HCQ and Tet on 4T1 cells and their lysosomal alkalinizing effects.

Previous studies have reported that cells with autophagy defects can activate macropinocytosis,^[^ ^29^, ^30^ ^]^ a pathway that allows tumor cells to take up nutrients from extracellular sources, resulting in the formation of numerous cytoplasmic vacuoles which then fuse with lysosomes for degradation and energy production. However, excessive macropinocytosis can lead to vacuolar accumulation and expansion, ultimately causing cell rupture and inducing macrophage‐like cell death.^[^ ^31^ ^]^ Therefore, the combination therapy of damaging mitochondria, inhibiting mitophagy, and inducing methuosis could potentially achieve a triple strike against cancer cells and merits further exploration.

Based on this, we designed and developed a type of nanoparticle (MTCAC NPs) for precise targeting of mitochondria and lysosomes, blocking the autophagy flux and progressively disrupting the internal environmental balance of cancer cells to kill tumors.

**Scheme 1:**
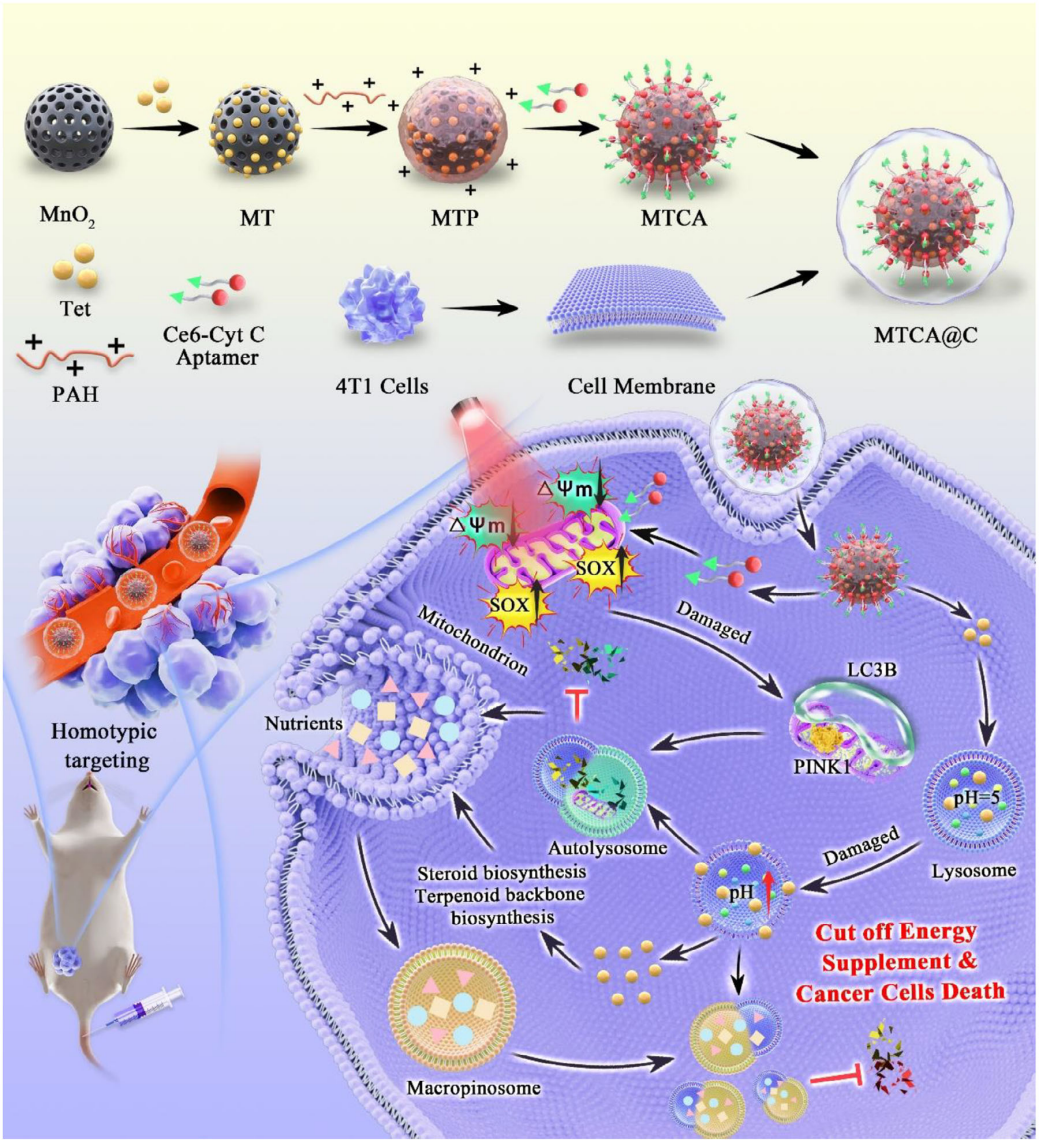
Schematic illustration of the smart NPs for synergistic tumor therapy. Mitophagy and macropinocytosisis were inhibited, leading to the occurrence of methuosis via dual disruption of mitochondria and lysosomes.

In this study, MTCA@C NPs can accumulate in tumors and responsively release Ce6‐Cyt C Apt and Tet in an acidic environment. Under near‐infrared light irradiation, Ce6 undergoes photodynamic reaction within mitochondria, producing a large amount of ROS to damage mitochondria. Tet alkalizes lysosomes, leading to their inability to degrade mitochondria normally and blocking the process of autophagy flux. The disruption of autophagic degradation and metabolic disorder turns into macropinocytosis to stimulate cells to absorb extracellular substances to provide nutrients as compensation. After lysosomal damage, the accumulation and expansion of macropinocytic vesicles that cannot be degraded eventually leads to the death of cancer cells under triple attack. Therefore, damaging mitochondria, blocking autophagy flux combined with blocking macropinocytosis triple hits on cancer cells provides a new therapeutic approach for the treatment of triple‐negative breast cancer (**Scheme** **1**).

## 2 Results and Discussion

### 2.1 Preparation and Characterization of MTCA@C NPs

**Figure 1:**
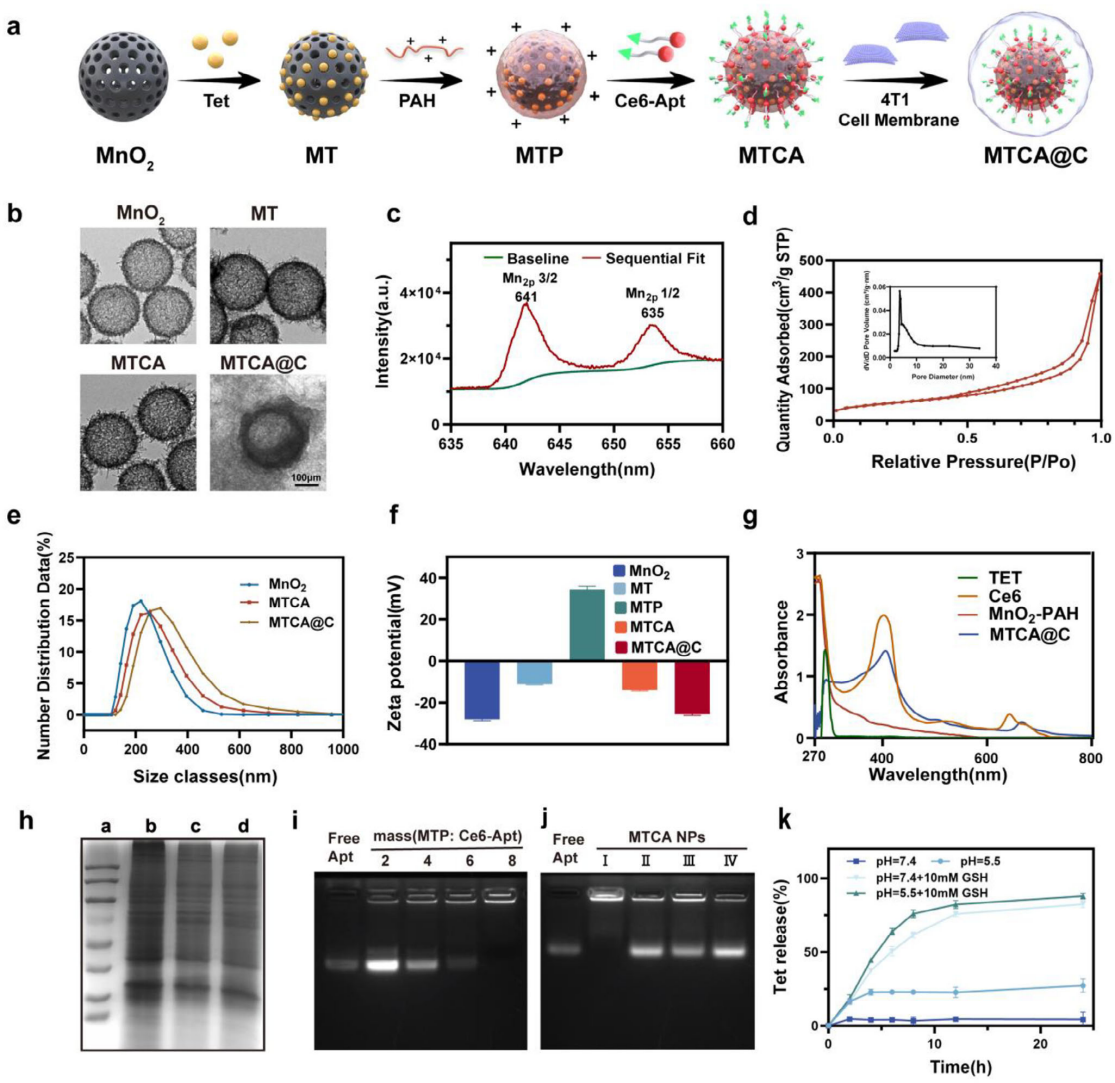
Preparation and characterization of MTCA@C NPs. a) Schematic diagram illustrating the stepwise synthesis of MTCA@C NPs. b) Representative transmission electron microscopy (TEM) images of different NPs. c) X‐ray photoelectron spectroscopy (XPS) spectrum of H‐MnO_2_ NPs. d) BET analysis and pore size distribution (inset) of H‐MnO_2_ NPs. e) DLS profiles of MnO_2_, MTCA, and MTCA@C NPs. f) Zeta potential measurements of different NPs. g) UV‐Vis‐NIR absorption spectra of different NPs. h) SDS‐PAGE analysis of whole proteins. (a:Marker; b:cell lysate; c:CM; d:MTCA@C). i) Agarose gel electrophoresis comparing MTP NPs loaded with Ce6‐Apt to the free aptamer. j) Agarose gel electrophoresis testing the release of Ce6‐Apt from MTCA@C under various conditions. (I:pH=7.4; II:pH = 7.4+10 mm GSH; III:pH = 5; IV:pH= 5+10 mm GSH). k) Release profile of Tet from MTCA@C (1 mg mL^−1^) under various conditions.

In this study, H‐MnO_2_ was synthesized using a method reported in prior literature.^[^ ^32^ ^]^ Analytical results demonstrated that the Ce6‐Aptamer (Ce6‐Apt) possesses >95% purity and correct sequence (Figures S1 and S2, Supporting Information). And the molecular weight of Ce6‐Apt was 13127.7 Da.Tet was loaded into the pores of H‐MnO_2_ via physical adsorption to from MnO_2_‐Tet (MT). Subsequently, a positively charged MnO_2_‐Tet/PAH (MTP) was created by PAH onto the outer layer of MT. Following this, negatively charged Ce6‐Apt was electrostatically adsorbed onto the outer layer of MTP to yield MnO_2_‐Tet/PAH/Ce6‐Apt (MTCA). Finally, MnO_2_‐Tet/PAH/Ce6‐Apt@CM (MTCA@C) NPs were produced through co‐extrusion with the 4T1 cell membrane using a polycarbonate membrane (**Figure** **1**a). TEM images revealed that the synthesized H‐MnO_2_ had a diameter of approximately 200 nm, featuring a uniform spherical morphology and mesoporous structure. The MTCA@C NPs exhibited a distinct core‐shell structure, indicating successful coating with the 4T1 cell membrane (Figure 1b). To further validate the composition of H‐MnO_2_, XPS was employed to determine the main elemental composition (Figure 1c and Figure S3, Supporting Information). Characteristic peaks at 653 and 641 eV corresponded to the Mn (IV) 2p3/2 and Mn (IV) 2p1/2 spin‐orbit peaks, respectively, confirming the +4 oxidation state of manganese in the NPs. The nitrogen adsorption isotherm demonstrated a clear hysteresis loop with an average pore diameter of 3.65 nm, indicating that the prepared MnO_2_ possessed a mesoporous structure and a large specific surface area, facilitating subsequent drug loading (Figure 1d).

DLS results showed a slight increase in the diameter of MTCA@C, with a hydrated particle size of approximately 290.5 ± 6.2 nm, confirming the successful coating of the 4T1 cancer cell membrane on the surface of MTCA NPs (Figure 1e). Zeta potential measurements indicated that after the adsorption of PAH onto the outer layer of MT, the charge shifted from ‐10 to +30 mV, suggesting successful electrostatic adsorption. Following the addition of negatively charged Ce6‐Apt, the charge shifted to ‐13 mV, confirming successful adsorption of Ce6‐Apt onto the MTCA NPs. After encapsulating the 4T1 cell membranes, the zeta potential of MTCA@C NPs was measured at ‐25.5 ± 0.7 mV (Figure 1f).

UV‐Vis spectroscopy revealed that the synthesized MTCA@C exhibited absorption peaks at 300, 402, and 662 nm, with a red shift at the 662 nm peak (Figure 1g), indicating successful encapsulation of both Ce6‐Apt and Tet within the MTCA@C NPs. To further validate the successful application of the cancer cell membrane coating, gel electrophoresis was conducted to analyze the protein components. There were no significant differences in the protein profiles between MTCA@C and the 4T1 cell membranes, demonstrating that the proteins on the 4T1 cell membranes were preserved during the preparation process, thus providing potential for homologous targeting of cancer cells (Figure 1h).

To maximize the Tet loading within the NPs, Tet was loaded into H‐MnO_2_ at varying mass ratios. The drug loading capacity peaked at approximately 80% at a 4:1 ratio (Figure S4, Supporting Information). To enhance the adsorption of Ce6‐Apt onto the MTP surface, a gel retardation assay was performed, showing that Ce6‐Apt could be completely adsorbed onto the MTP surface at an MTP‐to‐Apt ratio of 8:1 (Figure 1i). In vitro experiments simulating the tumor microenvironment were conducted to evaluate the drug release characteristics. And the results indicated that when MTCA@C was exposed to an environment with pH 5.5 and 10 mM GSH, substantial amounts of Ce6‐Apt and Tet were released, achieving release rates of 80% and 88% at 12 h and 24 h, respectively. In contrast, no significant release of Ce6‐Apt or Tet occurred at pH 7.4 (Figure 1j,k). Ce6‐Apt release from MTCA@C NPs exhibits minimal batch‐to‐batch variation across storage periods, demonstrating excellent short‐term stability (Figure S5, Supporting Information). In conclusion, a nanoplatform has been constructed that enables responsive release within the tumor microenvironment.

### 2.2 Cellular Uptake and Anticancer Effects of MTCA@C NPs

**Figure 2:**
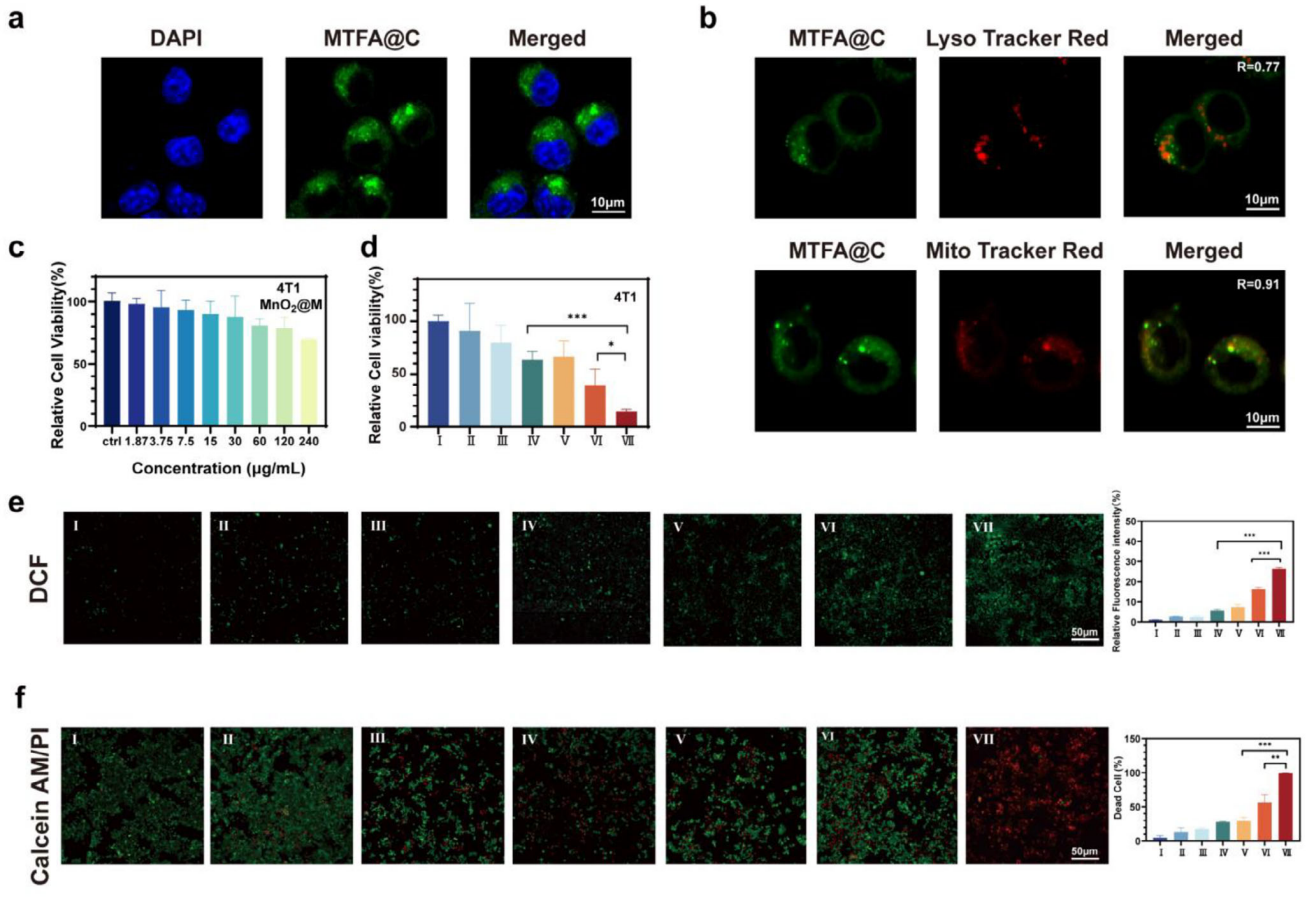
Cellular uptake and anticancer effects of MTCA@C NPs. a) Fluorescence image of 4T1 cells following treatment with MTCA@C NPs (scale bar: 10 µm). b) Colocalization of Lyso‐Tracker Red and Mito‐Tracker Red probes after overnight co‐incubation of 4T1 cells with MTCA@C NPs (scale bar: 10 µm). c) CCK‐8 assay results for 4T1 cells treated with varying concentrations of MnO_2_@CM (scale bar: 10 µm). d) CCK‐8 assay results for 4T1 cells subjected to different treatments. e) Representative fluorescence images of DCFH‐DA‐stained 4T1 cells, analyzing intracellular ROS levels after various treatments. Quantitative analysis of green fluorescence intensity is shown (scale bar: 50 µm). f) Representative fluorescence images of 4T1 cells under different treatments. Green indicates Calcein‐AM (live cells) and Red indicates PI (dead cells). Quantitative analysis of red and green fluorescence intensity is presented (scale bar: 50 µm). (I: PBS; II: Ce6; III: Tet; IV: MT@C; V: MC@C; VI: MCA@C; VII: MTCA@C). All data are presented as the mean ± SD (*p < 0.05; * *p* < 0.05, **p < 0.01; ***p < 0.001; two‐tailed Student's *t*‐tests).

To evaluate the uptake capability of 4T1 cells for MTCA@C NPs, FAM was employed as a fluorescent probe linked to aptamer to create MTFA@C NPs. After 6 h of incubation, bright green fluorescence was observed in the cytoplasm of 4T1 cells, indicating that the NPs could be internalized by these cells (**Figure** **2**a). Furthermore, the subcellular localization of the NPs was investigated. As shown in the images, the colocalization coefficients of MTFA@C NPs with lysosomes and mitochondria were 0.77 and 0.91, respectively, suggesting that the NPs successfully escaped from lysosomes before reaching the mitochondria (Figure 2b).

Since the biosafety of nanocarriers is a crucial factor in biomedical applications, the toxicity of MnO_2_/CM in 4T1 cells was also assessed. The results indicated that cytotoxicity was negligible at the tested concentrations (Figure 2c). The CCK‐8 assay results demonstrated that the anti‐cancer effect of the complete MTCA@C system was significantly stronger than that of the MT and MCA monotherapy groups, achieving a cancer cell killing rate of 85% (Figure 2d). To detect ROS generation, DCFH‐DA was utilized as a fluorescent probe. The results showed that ROS levels in the MTCA@C group were significantly elevated compared to the other groups, being 4.3 times and 3.7 times higher than those in the MT@C and MCA@C groups, respectively (Figure 2e). Moreover, calcein‐AM/PI fluorescence staining to assess the live/dead status of 4T1 cells corroborated these findings (Figure 2f).

These findings confirm that MTCA@C NPs can escape from lysosomes and reach mitochondria, thereby inducing cytotoxic effects through photodynamic reactions.

### 2.3 Mitochondrial Damage of MTCA@C NPs

**Figure 3:**
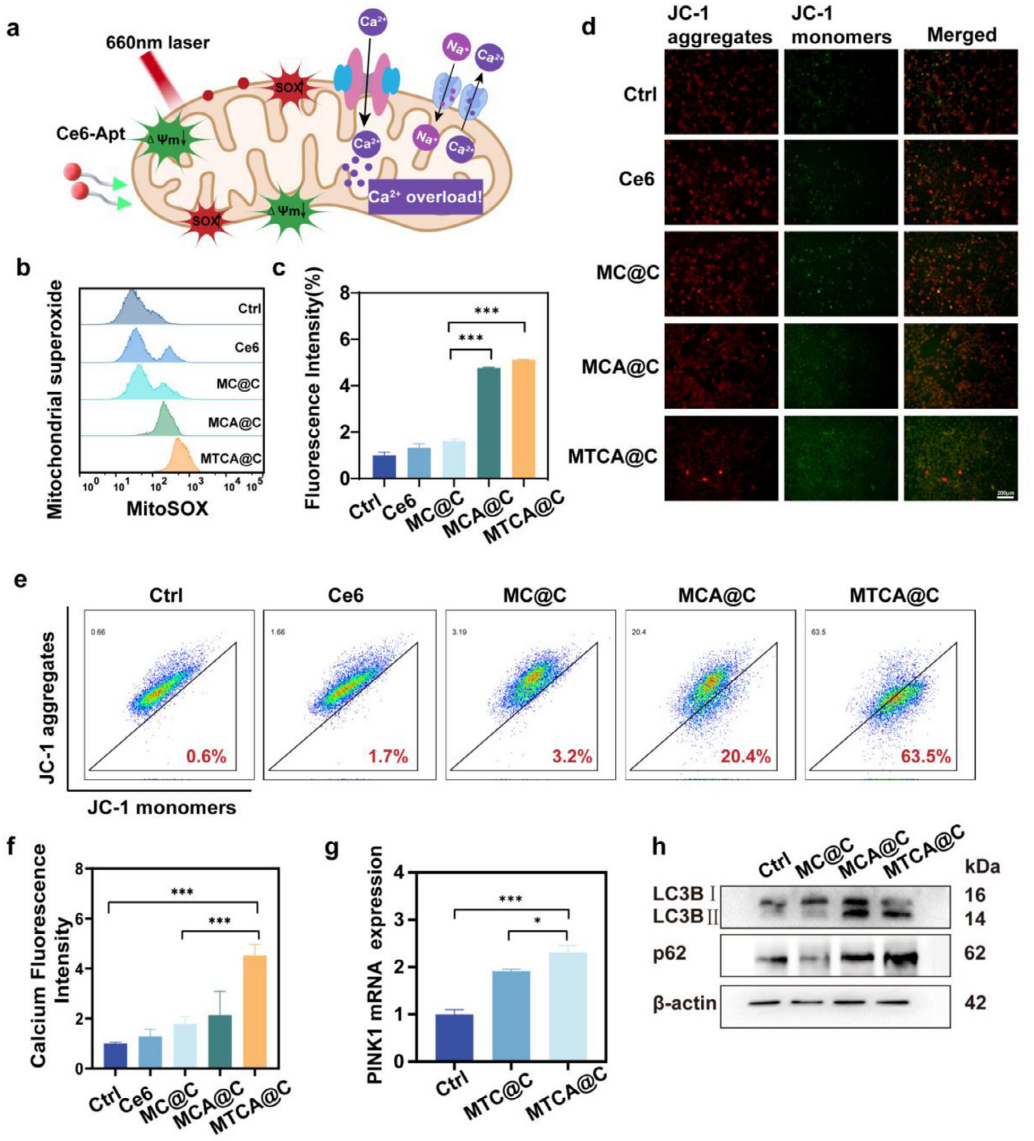
Mitochondrial damage induced by MTCA@C NPs. a) Schematic representation of the mechanism by which Ce6‐Apt induce mitochondrial damage in 4T1 cells. b) Flow cytometry analysis of 4T1 cells to detect mitochondrial superoxide fluorescence following treatment with different NPs. c) Quantitative analysis of mitochondrial superoxide fluorescence intensity. d) JC‐1 staining images of 4T1 cells. (scale bar: 200 µm). e) Flow cytometry results for JC‐1 assay. f) Fluorescence intensity of calcium ion dye in 4T1 cells after various treatments. g) Real‐time qPCR analysis of PINK1 levels in 4T1 cells treated with different NPs. (h) Western blot analysis of LC3B II and p62 expression. All data are presented as the mean ± SD (*p < 0.05; **p < 0.01; ***p < 0.001; two‐tailed Student's *t*‐tests).

The results above demonstrate that MTCA@C NPs can specifically target mitochondria due to the aptamer linked to Ce6, which can recognize cytochrome C within the mitochondria. Consequently, upon exposure to near‐infrared light, a photodynamic reaction occurs inside the mitochondria with Ce6, resulting in mitochondrial damage (**Figure** **3**a). The generation of superoxide within the mitochondria was assessed using MitoSOX Red dye, which revealed that the MCA@C group, featuring the aptamer, produced significantly more superoxide compared to the MC@C group without the aptamer, showing a 2.9 times increase (Figure 3b,c). Mitochondrial damage is also evidenced by a decrease in mitochondrial membrane potential (MMP). The MMP following various treatment regimens was evaluated using the JC‐1 probe, and the results indicated a prominent increase in green fluorescence and a marked decrease in red fluorescence in the MTCA@C group (Figure 3d and Figure S6, Supporting Information). Flow cytometry analysis corroborated these findings, showing that both the MCA@C and MTCA@C groups exhibited a reduction in MMP compared to the PBS group. Notably, the decrease in MMP was most pronounced in the MTCA@C group, which showed a reduction of 63.5% (Figure 3e). Additionally, intracellular calcium ion concentrations were measured. After treatment with MTCA@C NPs, the intracellular calcium levels were 4.5 times higher than those of the control group and 2.5 times higher than those of the MC@C group (Figure 3f), suggesting that mitochondrial damage induces calcium efflux, leading to calcium overload within the cells.

When mitochondrial damage occurs, mitophagy is spontaneously activated as a protective mechanism. The PTEN‐induced kinase 1 (PINK1)/Parkin pathway plays a crucial role in the removal of damaged mitochondria. When the mitochondrial membrane potential is compromised, the pathway for PINK1 to enter the inner mitochondrial membrane is disrupted, resulting in increased expression of PINK1 on the outer mitochondrial membrane. The expression of PINK1 mRNA in cells was measured using qRT‐PCR (Figure 3g). The results indicated that the PINK1 mRNA levels in the MCA@C group increased 2.3 times compared to the control group and 1.3 times compared to the MC@C group. This suggests that oxidative stress within the mitochondria activates the PINK1/Parkin pathway, thereby inducing mitophagy.

Subsequently, the expression of autophagy‐related proteins was evaluated via Western blot analysis (Figure 3h and Figure S7, Supporting Information). Upon the activation of cellular autophagy, the LC3 I is converted to the autophagosomal membrane‐associated LC3 II. The elevated expression of LC3 II observed in both the MCA@C and MTCA@C groups indicates a significant increase in the number of autophagosomes, suggesting that autophagy was activated in tumor cells as a defensive response to perceived stress. Importantly, p62, a protein selectively recruited to the autophagosomal membrane for autolysosomal degradation, exhibited significant accumulation in the MTCA@C group. This accumulation suggests that the autophagic degradation processes were impeded.

In summary, this section confirms that MTCA@C NPs specifically target mitochondrial damage, activating the PINK1/Parkin pathway, which subsequently leads to the occurrence of mitophagy.

### 2.4 Lysosomotropic Activity and Inhibition of Autophagy of MTCA@C NPs

**Figure 4:**
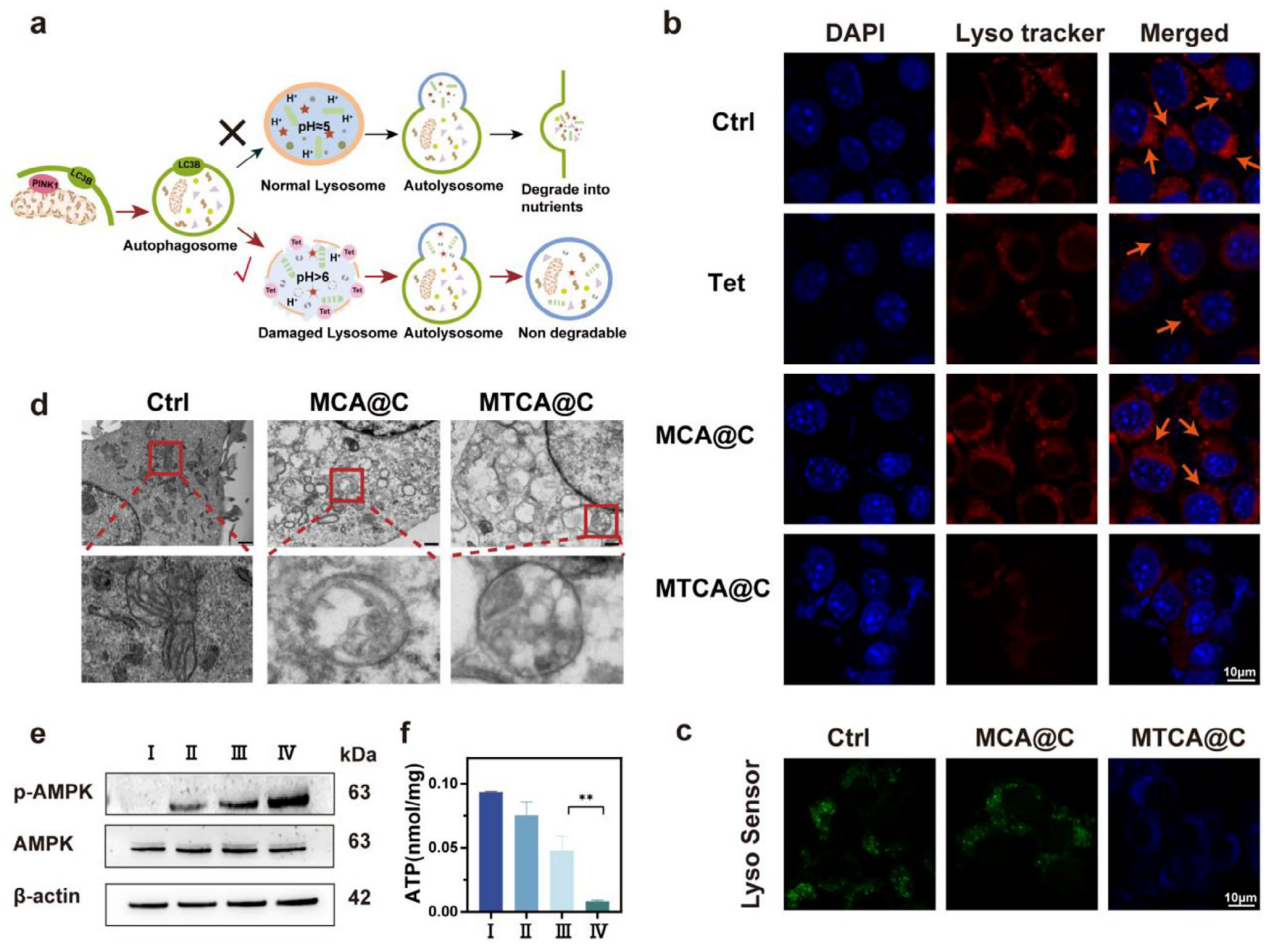
Alkalization of lysosomes and inhibition of autophagy by MTCA@C NPs. a) Schematic diagram of the mechanisms of autophagy inhibition. b) Fluorescence pattern of 4T1 cells after staining with lysosomal dyes after different treatments (scale bar: 10 µm). c) Confocal laser scanning microscopy images of 4T1 cells treated with various NPs and stained with LysoSensor Yellow/Blue DND‐160 (scale bar: 10 µm). d) Bio‐transmission electron microscopy (Bio TEM) images of cells treated with PBS, MCA@C, and MTCA@C NPs (scale bar: 500 nm). e) Western blot analysis showing the expression levels of AMPK and phosphorylated AMPK (p‐AMPK). f) ATP concentration in 4T1 cells subjected to different treatments (I: PBS, II: Tet, III: MCA@C, IV: MTCA@C). The data are presented as the mean ± SD. (n=3, *p < 0.05, ** *p* < 0.01, *** *p* < 0.001; two‐tailed Student's *t*‐tests).

HCQ has been used as an autophagy inhibitor in multiple studies and is known to alkalinize lysosomes. However, HCQ exerts its autophagy‐inhibiting effects only at higher concentrations. Tet is also reported to alkalinize lysosomes. We compared the lysosomal alkalinization effects of HCQ and Tet using the LysoSensor Yellow/Blue DND‐16 dye at two different concentrations. The results showed that at a dosage of 10 µg mL^−1^, there were differences in the alkalizing effects of the two drugs on lysosomes. Also Tet exhibited a more significant cytotoxic effect on 4T1 cells at a lower dose(20 µg mL^−1^) (Figure S8, Supporting Information). Therefore, Tet was selected as the lysosomal alkalizer (**Figure** **4**a).

After drug accumulation in the lysosome, we compared the lysosomal function across the treatment groups. The principle of the red lysosomal tracker probe is based on the acidity of the lysosomes. After 6 h of incubation at 10 µg mL^−1^, the lysosomal red spots in the MTCA@C‐treated cells were significantly fewer than those in the Tet‐treated cells (Figure 4b). This result indicates that MTCA@C can increase the lysosomal pH and cause lysosomal dysfunction, with MTCA@C NPs causing more severe lysosomal damage compared to free Tet. Moreover, cells treated with MTCA@C NPs exhibited signs of cell death, such as nuclear condensation and dissolution. Additionally, we treated the cells with the LysoSensor Yellow/Blue DND‐160 dye, revealing that lysosomes in the MCA@C group were stained green, while those in the MTCA@C group were blue, further confirming the alkalinizing effect of Tet on lysosomes (Figure 4c).

The aforementioned experiments validated that Tet can damage lysosomes, preventing them from fully degrading autophagosomes, which was further corroborated by bio‐TEM (Figure 4d). The control group showed normal mitochondrial morphology, with orderly arranged cristae. In contrast, mitochondria in the MCA@C group exhibited blank areas, swelling, and broken cristae, along with autophagosomes enveloping the mitochondria with double membranes, indicating that MCA@C can damage mitochondria and induce autophagy. Similarly, mitochondria in the MTCA@C group also displayed blank areas, swelling, and broken cristae, but additionally exhibited autophagic lysosomes with single membranes, suggesting that Tet‐induced lysosomal damage may prevent the degradation of autophagosomes. Furthermore, we validated the activation of the AMPK pathway via western blotting (Figure 4e and Figure S9, Supporting Information), which showed a 5.6 times increase in p‐AMPK protein expression in the MTCA@C group compared to the control group, indicating activation of the AMPK pathway and suggesting a potential energy deficiency signal in cancer cells. We then used an ATP detection kit to measure ATP production in the cells, revealing that the MTCA@C group produced the least ATP, while the MCA@C group produced 5.0 times more ATP, and the control group produced 11.2 times more, indicating that MTCA@C most effectively blocks the energy supply in cancer cells. The addition of Tet significantly disrupts the autophagic flux that supplies nutrients to cancer cells (Figure 4f).

### 2.5 MTCA@C NPs Induce Macropinocytosis and Methuosis

**Figure 5:**
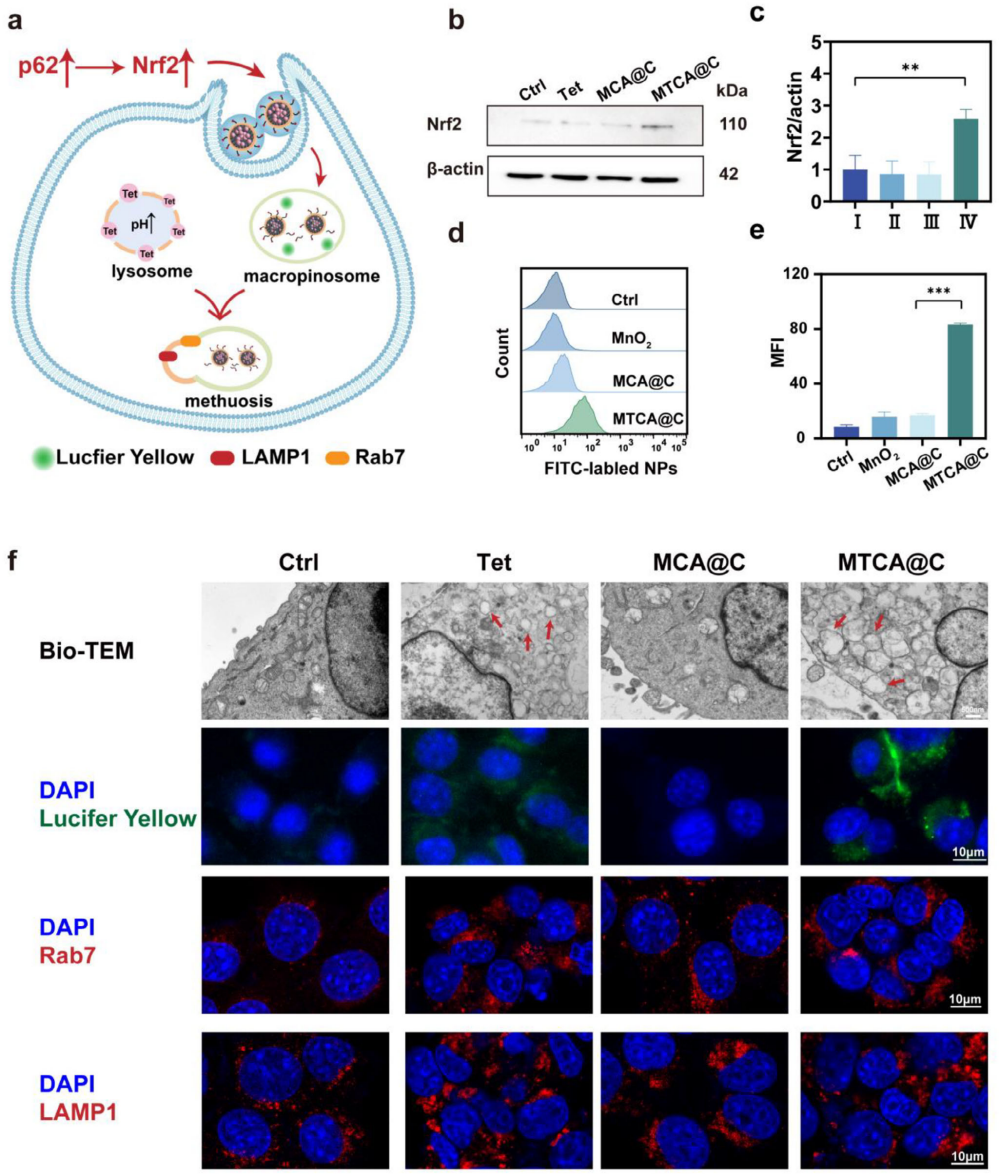
MTCA@C NPs induce macropinocytosis and methuosis. a) A diagram of how macropinocytosis turns into methuosis. b) Western blot analysis of NRF2 expression levels. c) Grayscale analysis of western blotting of NRF2/β‐actin. d) Flow cytometric analysis results of 4T1 cells treated with various NPs. e) Quantitative analyses of fluorescence intensity treated by different nanoparticles. f) Bio‐TEM images of 4T1 cells (scale bar: 500 nm) and images of living 4T1 cells treated with Lucifer Yellow dye (scale bar: 10 µm). Immunofluorescent staining of RAB7 and LAMP1 in 4T1 cells treated with PBS, Tet, MCA@C, and MTCA@C NPs (scale bar: 10 µm). All data are presented as the mean ± SD (*n* = 3; *p < 0.05, **p < 0.01; ***p < 0.001; two‐tailed Student's *t*‐tests).

Previous studies have found that autophagy inhibition may lead to the transition of cells to macropinocytosis to uptake extracellular materials, compensating for the loss caused by impaired autophagy (**Figure** **5**a). Given that NRF2 is a central transcriptional activator of macropinocytosis, western blotting results showed that the expression of NRF2 protein in the MTCA@C group was upregulated, with a level 2.4 times higher than that of the control group and 4.5 times higher than that of the MCA@C group (Figure 5b,c). This suggests that cancer cells may engage in macropinocytosis to re‐acquire nutrients from the environment, which is associated with the upregulation of NRF2. Consequently, we investigated the signaling pathways related to macropinocytosis. Flow cytometry results indicated that the MTCA@C group had the highest uptake of NPs labeled by FITC, reaching 4.8 times that of the MCA@C group (Figure 5d,e). This suggests that when autophagic flux is blocked in cancer cells, macropinocytosis is activated, prompting cells to uptake more NPs.

Additionally, TEM results revealed that both the Tet and MTCA@C treatment groups exhibited a significant accumulation of vacuoles in the cytoplasm (Figure 5f), which is consistent with the intracellular structural changes associated with macropinocytosis.^[^ ^32^ ^]^ A typical feature of macropinocytosis is the presence of vacuoles that can incorporate extracellular fluid tracers. Lucifer Yellow dye, a stable tracer for fluid‐phase endocytosis, was used for live‐cell imaging. The results showed the accumulation of Lucifer Yellow in the transparent vacuoles formed in the Tet and MTCA@C treated cells, similar to findings reported in previous literature (Figure 5f and Figure S10, Supporting Information). Since vacuoles in macropinocytosis are characteristic of late endosomes (RAB7 and LAMP1 positive). Immunofluorescence showed that the expression levels of RAB7 and LAMP1 were up‐regulated in both Tet and MTCA@C groups (Figure 5f and Figure S10, Supporting Information). The above data confirmed that macropinocytosis was activated in the whole drug group.

Additionally, an intriguing observation was made: there was no significant change in Nrf2 protein levels within the cells after Tet treatment. However, both electron microscopy and fluorescence experiments demonstrated that cells treated with Tet also underwent macropinocytosis. Therefore, we proceeded to investigate how Tet activates cells to engage in macropinocytosis.

### 2.6 Gene Expression Analysis

**Figure 6:**
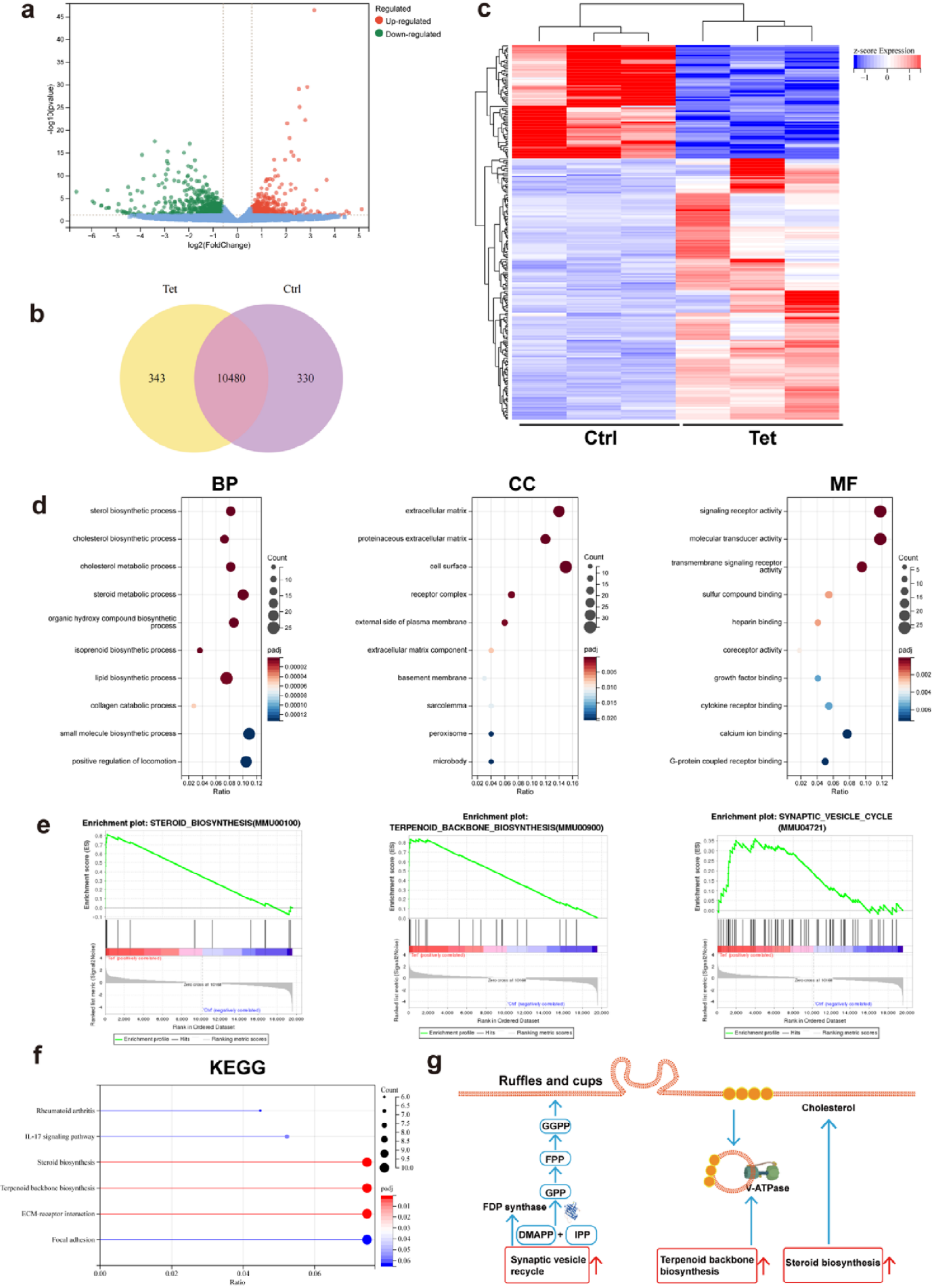
Gene expression analysis in 4T1 cells under two treatment condition. a) The volcano plot illustrates the differentially expressed genes (DEGs) in 4T1 cells treated with PBS compared to those treated with Tet. b) Venn diagrams. c) Heatmap of upregulated and downregulated genes in cell samples treated with PBS and Tet (*n* = 3). d) Gene Ontology (GO) enrichment analyses were performed for DEGs unique to each treatment group, categorizing them into biological processes (BP), cellular components (CC), and molecular functions (MF). e) Gene Set Enrichment Analysis (GSEA) was conducted to evaluate gene sets related to macropinocytosis in both treatment groups, with ES representing the enrichment score and NES indicating the normalized enrichment score. f) Kyoto Encyclopedia of Genes and Genomes (KEGG) enrichment analyses were performed for DEGs unique to the combination treatment group. g) An illustration depicts the mechanism by which macropinocytosis is activated, as determined by RNA sequencing (RNA‐seq) analysis.

Through RNA sequencing, a volcano plot was generated to provide a fundamental description of the differentially expressed genes (DEGs) between the control and Tet samples. We identified 72 upregulated and 164 downregulated DEGs in the Tet group (**Figure** **6**a–c), with the heatmap illustrating the changes in gene expression. Notably, the “sterol biosynthesis” pathway ranked first in both biological processes (BP) and Kyoto Encyclopedia of Genes and Genomes (KEGG) enrichment analyses (Figure 6d,f). Cholesterol, a vital component of all biological cell membranes, plays a critical role in maintaining the normal physiological function and fluidity of the cell membrane. The transcriptional upregulation of the “steroid biosynthesis” pathway is hypothesized to result from the activation of macropinocytosis, leading to the formation of large and irregular endocytic vesicles due to membrane folding—a process that necessitates the consumption and replenishment of cholesterol.^[^ ^33^ ^]^ This finding emphasizes the significant relationship between Tet and cholesterol synthesis.

Furthermore, Gene Set Enrichment Analysis (GSEA) of the 72 upregulated DEGs revealed associations with the “terpenoid backbone biosynthesis” and “synaptic vesicle cycle” pathways (Figure 6e). The most prominently upregulated gene was Fdps, a key enzyme in isoprenoid biosynthesis. This enzyme facilitates the consecutive condensation of dimethylallyl pyrophosphate, ultimately producing geranyl geranyl pyrophosphate (GGPP), which promotes membrane folding and the formation of cup‐shaped structures that facilitate macropinocytosis.^[^ ^34^, ^35^ ^]^ The qRT‐PCR experiment confirmed that the transcription of the Fdps gene was significantly upregulated in the 4T1 cells treated with Tet (Figure S11 and Table S2, Supporting Information). Additionally, the transcriptional upregulation of V‐ATPase, which promotes vesicle recycling, further supports the conclusion that Tet can enhance macropinocytosis (Figure 6g).

### 2.7 Tumor Targeting of MTCA@C NPs In Vivo

**Figure 7:**
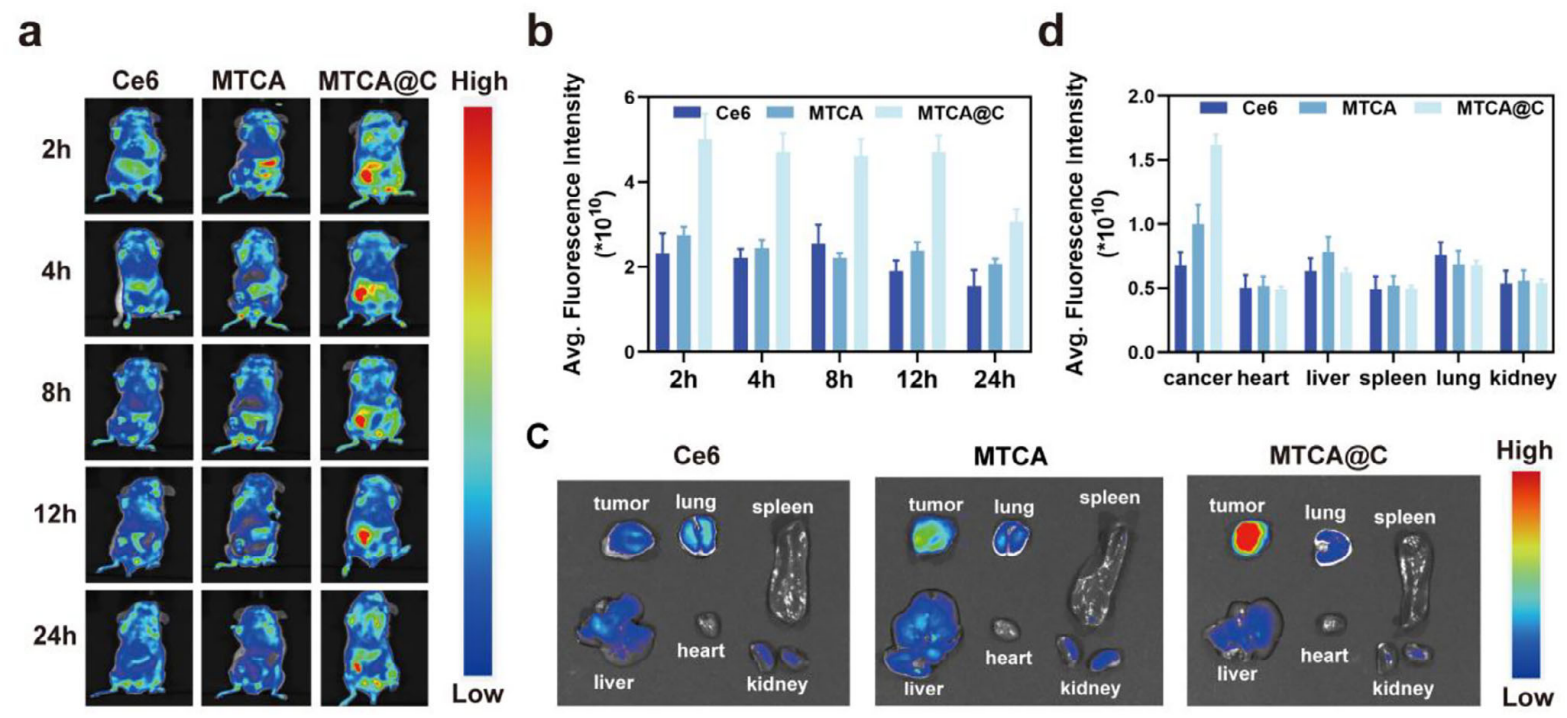
Tumor targeting of MTCA@C NPs in vivo. a) Time‐dependent fluorescence imaging of 4T1 tumor‐bearing BALB/c mice and treated with Ce6, MTCA and MTCA@C NPs. b) Quantitative analyses of tumor fluorescence intensity measurements were shown (*n* = 3). c) Biodistribution of Ce6, MTCA and MTCA@C NPs in different major organs at 24 h. d) Quantitative analyses of major organ fluorescence intensity measurements were shown (*n* = 3).

The MTCA@C group which was encapsulated with 4T1 cell membranes, exhibited more pronounced accumulation compared to the other two groups (**Figure** **7**a). To quantitatively evaluate the tumor targeting efficiency of MTCA@C NPs, mice were dissected 24 h post‐injection, and in vitro imaging was also performed. Among them, the tumor signal in the MTCA@C group was the strongest, being 1.5 times that of MTCA group and 3.0 times that of Ce6 group (Figure 7b). Meanwhile, the NPs mainly accumulated at the tumor site, while only a small amount retained in non‐target areas through the bloodstream (Figure 7c,d). These results suggested that the external coating of homologous cell membranes on MTCA@C NPs can enhance their tumor targeting and accumulation, thereby reducing the possibility of non‐specific elimination.

### 2.8 In Vivo Therapeutic Efficacy

**Figure 8:**
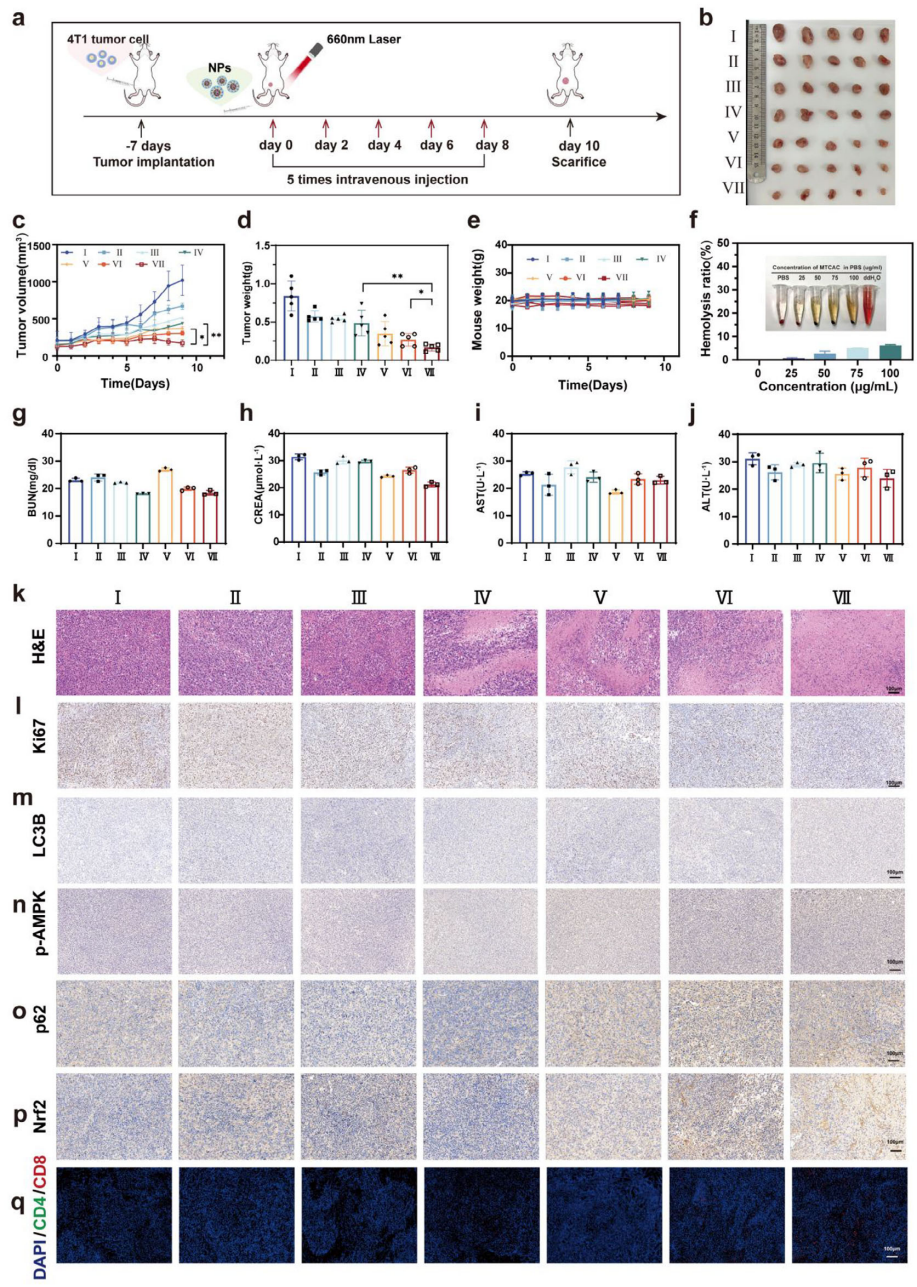
In vivo therapeutic efficacy. a) Experimental protocol for in vivo antitumor treatment. b) Photographs of excised tumors following the final administration. c) Tumor growth curves for different treatments. d) Changes in tumor weight of mice across various treatment groups. e) Changes in weight of mice across various treatment groups. f) Assessment of hemolysis in red blood cells incubated with different concentrations of MTCA@C NPs for 6 h. g–j) Blood biochemical analyses of serum from different treatment groups, including measurements of blood urea nitrogen (BUN), creatinine (CREA), aspartate aminotransferase (AST), and alanine aminotransferase (ALT) levels. k) Representative H&E staining. (Scale bar, 100 µm). l–p) Representative IHC analysis of Ki67, LC3B, p‐AMPK, p62 and Nrf2 in tumor tissues. (Scale bar, 100 µm). q) Immunofluorescence staining of CD4^+^/CD8^+^ in tumor tissues. (Scale bar, 100 µm). (I: PBS; II: Ce6; III: Tet; IV: MT@C; V: MC@C; VI: MCA@C; VII: MTCA@C). All data are presented as the mean ± SD (*p < 0.05; **p < 0.01; ***p < 0.001; two‐tailed Student's *t*‐tests).

The therapeutic efficacy of the nanodrug in vivo was further analyzed using 4T1 tumor‐bearing mice that were randomized into seven groups, each treated with different formulations via intravenous administration when the tumor volume reached approximately 100 mm^3^ (**Figure** **8**a). Throughout the treatment, tumor volumes in all experimental groups were monitored, and tumor tissues were harvested and weighed after euthanasia. The results indicated that the tumor tissue in the MTCA@C group was significantly smaller than that in the other groups (Figure 8b), and the tumor weight in the MTCA@C group was considerably lighter compared to the other groups (Figure 8c). The data on tumor volumes across all experimental groups further confirmed the superior tumor inhibition efficacy of MTCA@C NPs (Figure 8d).

However, the biosafety of these synergistic therapeutic NPs remains a primary concern in clinical settings. Daily monitoring of the mice's body weight showed no significant differences among the groups (Figure 8e). Additionally, major organs from each group were collected for analysis after the treatment concluded. H&E staining results revealed no obvious pathological features distinguishing the PBS control group from the treatment groups (Figure S12, Supporting Information). There was no hemolysis observed in the MTCA@C group at varying concentrations after 6 hours of continuous exposure (Figure 8f). Moreover, the indicators showed no statistically significant differences between the PBS and treatment groups, indicating that the NPs exhibited no significant nephrotoxicity or hepatotoxicity in mice (Figure 8g–j). H&E staining of the bilateral tumors further confirmed the superior tumor‐suppressive efficacy of the MTCA@C group (Figure 8k). Immunohistochemical analysis of Ki67 revealed a significant reduction in the number of Ki67‐positive cells within the tumor tissue after MTCA@C injection, with a positive rate of 53.4%, markedly lower than that observed in the other groups (Ce6: 92.2%, Tet: 75.6%, M‐T: 68.2%, MC@C: 62.1%, MCA@C: 58.7%) (Figure 8l and Figure S13, Supporting Information). Additionally, the immunohistochemical results for LC3B indicated elevated mitophagy levels in the mice of the MTCA@C group, approximately 2.8 times that of the MT@C group and 2.1 times that of the MC@C group (Figure 8m and Figure S14, Supporting Information). The expression of p‐AMPK protein in the MTCA@C group increased by 2.9 times compared to the MT group and 1.9 times compared to the MC@C group (Figure 8n and Figures S15 and S16, Supporting Information). Compared with their respective control groups, the protein expression of p62 and Nrf2 in tumor tissues, was elevated to 2.5‐fold and 2.7‐fold, respectively (Figure 8o,p and Figures S17 and S18, Supporting Information).

Previous studies have shown that Mn^2+^ generated by MnO_2_ degradation can activate the cGAS‐STING pathway; therefore, western blotting experiments were conducted to verify the expression of STING and p‐STING proteins. The results indicated that the expression of p‐STING protein in the MTCA@C group was approximately 3.0 times higher than that in the control group, confirming the activation of the STING pathway (Figure S19, Supporting Information). Furthermore, immunofluorescence results demonstrated that the MTCA@C group generated the highest number of CD8^+^ T cells, indicating that the MTCA@C treatment possibly activated the antitumor immune response in mice (Figure 8q and Figure S20, Supporting Information). Notably, the MTCA@C NPs, which can effectively disrupt cancer cells energy supply via triple mechanisms, exhibited remarkable antitumor effects.

The triple‐mechanism action, which disrupts both mitochondria and lysosomes while inducing methuosis, provides a more comprehensive energy blockade, potentially overcoming compensatory resistance seen with single‐target therapies. The long‐term biocompatibility and potential immunogenicity of the biomimetic system upon repeated administration require further investigation.

## 3 Conclusion

In summary, we designed an intelligent responsive MTCA@C NPs that enhances homologous targeting and biosafety through cell membrane modification. The assembled NPs can accurately target the mitochondria of cancer cells via a cytochrome C aptamer. Upon irradiation with near‐infrared light, a photodynamic reaction takes place within the mitochondria, leading to severe mitochondrial dysfunction and an initial reduction in energy supply. Meanwhile, Tet functions as a lysosomal alkalizing agent, blocking autophagy, thereby severing the secondary energy source in cancer cells. Both autophagy deficiency and Tet can activate macropinocytosis, allowing for nutrient uptake from the extracellular environment to partially restore energy supply. However, due to the compromised function of lysosomes, a significant accumulation of vacuoles occurs within the cells, resulting in energy depletion and subsequent methuosis in cancer cells. This progressive strategy of cutting off energy supply pathways through triple mechanisms presents a novel approach to the treatment of breast cancer and provides a potential therapeutic strategy for repurposing Tet in oncology.

## 4 Experimental Section

### Materials

All chemical reagents were used directly without further purification. Triton X‐100, tetraethyl orthosilicate (TEOS), PAH and dopamine hydrochloride (DA) were brought from Aladdin (USA). Cyclohexane, ammonia, KMnO_4_, and NaOH were purchased from Sinopharm Chemical Reagent Co., Ltd. (China). Bovine serum albumin (BSA) obtained from Saiguo Biotech Co. Ltd. (China). Oligonucleotides without modification were ordered from Sangon Biotech. Co., Ltd. (Shanghai, China). And the chlorin e6‐Cyt C‐Aptamer (Ce6‐Apt) used in this work was synthesized and purified by Takara Biotechnology Co., Ltd. (Dalian, China). PI and calcein‐AM dye were purchased from Shanghai Sig Biotechnology Co., Ltd., (Shanghai, China). DAPI, Mito‐Tracker and Lyso‐Tracker were purchased from Beyotime Biotechnology Co., Ltd. (Shanghai, China). LC3B II (ET1701‐65), AMPK (ET1608‐40), p‐AMPK (ET1612‐72) and p62 (HA721171) antibodies were purchased from HUABIO Biotechnology Co., Ltd. (Hangzhou, China). LAMP1 (A24804PM) and RAB7 (A22400) antibodies were purchased from Abconal Biotechnology Co., Ltd. (Wuhan, China).

### Synthesis of H‐MnO_2_

H‐MnO_2_ was prepared according to previously described procedures.^[^ ^36^ ^]^ First, silica NPs (SiO_2_) were synthesized as templates. Trixon X‐100 (53 mL), cyclohexane (225 mL), n‐hexanol (54 mL), ammonia (7.5 mL), H_2_O (10 mL), and tetraethyl orthosilicate (5 mL) were added and stirred for 12 h. SiO_2_ NPs were collected by centrifugation (13000 rpm, 15 min) and washed with anhydrous ethanol and ddH2O twice. Then 123 mg SiO_2_ and 100 mg DA were added in 50 mL Tris buffer (10 mm, pH 8.5) and reacted for about 3 h at room temperature and centrifuged to collect SiO_2_@DA. Next, KMnO_4_ aqueous solution (2 mg mL^−1^, 30 mL) was dropped into SiO_2_@DA, stirred and reacted for 6 h, and the NPs were collected by centrifugation (13 000 rpm, 20 min). At last, hollow manganese dioxide (H‐MnO_2_) NPs were etched with NaOH solution (1 m) at 80 °C for 6 h.

### Isolation of 4T1 Cell Membrane

When 4T1 cells were 90% confluent, cells were collected and centrifuged (3000 rpm, 10 min, 4 °C) to obtain cell precipitation. Then, the cell precipitation was resuspended by hypotonic lysis buffer (2 mm MgCl_2_, 10 mm KCl, 1 m tris–HCl, 50 mL H_2_O) and transferred to a homogenizer to homogenize up and down 15 times, further centrifuged (6000 rpm, 10 min, 4 °C) to get the supernatant. Repeated the operation once, the supernatant was then ultracentrifuged (46 000 rpm, 60 min, 4 °C) to obtain the 4T1 cell membrane.

### Preparation of MTCA@C NPs

The H‐MnO_2_ was mixed with Tet at room temperature according to a specific mass ratio and stirred overnight. The MT NPs were then collected by centrifugation (13000 rpm, 10 min) and dispersed in ddH_2_O after two rounds of centrifugal washing with ddH_2_O. Subsequently, PAH solution was mixed with MT solution for 12 h at a mass ratio of 6:1. The resulting MTP NPs were collected by centrifugation. Then the Ce6‐Apt and MTP NPs were mixed for 30 minutes at a mass ratio of 1:8, and the MTCA NPs were also collected by centrifugation. Furthermore, the extracted 4T1 cell membrane was first passed through a 400 nm polycarbonate membrane to obtain empty cell membrane vesicles. After that those collected NPs were evenly mixed with vesicles at a mass ratio of 1:2, followed by co‐extrusion through 400 nm polycarbonate membranes in a lipid extrusion machine to obtain MTCA@C NPs and centrifuged to remove the empty vesicles.

### Characterization of MTCA@C NPs

The morphology of the NPs was examined using transmission electron microscopy (TEM). 10 µL sample solution was dropped onto a carbon‐coated copper mesh (300 mesh) and allowed to stand for several minutes. Subsequently negatively stained with 1% (v/v) uranyl acetate. The morphology was then observed under a TEM (Hitachi, Japan). Furthermore, the dynamic light scattering and zeta potential of the NPs were assessed utilizing the dynamic light scattering instrument (Malvern, UK). The ASAP2460 analyzer (Micromeritics, USA) was employed to analyze the nitrogen (N_2_) adsorption‐desorption isotherm of MTCA@C NPs, and the specific surface area and aperture were determined using the BET method.

### Cell Culture

The 4T1 cell (RRID:CVCL_0125) was obtained from the American Type Culture Collection (ATCC). The cells were routinely tested and confirmed to be free of mycoplasma contamination. The 4T1 cell was cultured in Dulbecco's modified Eagle's medium supplemented with 10% fetal bovine serum and 1% penicillin streptomycin in a humidified atmosphere of 95% air and 5% CO_2_ with the temperature of 37 °C.

### Western Blotting Analysis

Proteins were extracted from cells and denatured and stored after quantification. The proteins were then resolved by sodium dodecyl sulfate‐PAGE. Separated proteins were transferred to a polyvinylidene fluoride (PVDF) membrane and blocked with 5% fat free milk. The membrane was then incubated with primary antibodies at 4 °C overnight followed by horseradish peroxidase‐conjugated secondary antibodies for 2 h at room temperature. Finally, signals were observed using Tanon TM High‐sig ECL Western Blotting substrate.

### Intracellular Autophagy Detection

4T1 cells were treated with PBS, MCA@C (1 µg mL^−1^ Ce6 equiv), MTCA@C (1 µg mL^−1^ Ce6 equiv, 10 µg mL^−1^ Tet equiv) overnight, following by laser irradiation (660 nm, 3 min, 0.7 W cm^−2^) and incubated at 37 °C. Afterward, the cells were collected to obtain cell precipitation, then fixed with 2.5% glutaraldehyde at 4 °C. The morphology of intracellular mitochondria, autophagosomes, and autolysosomes was observed under transmission electron microscope (Hitachi, Japan).

### Immunofluorescence Staining

Cells were fixed with 4% (v/v) paraformaldehyde for 15 min and permeabilized with 0.1% Triton X‐100 for 20 min, followed by blocking with 5% BSA for 1 h. Then, the cells were incubated with RAB7 and LAMP1 antibodies overnight at 4 °C. After washing for three times with PBS, the cells were incubated with Alexa Fluor 594‐conjugated anti‐rabbit IgG for 1 h. Subsequently, the cells were stained with DAPI staining solution. All images were captured under fluorescence microscope (Leica, Germany)

### RNA Sequencing Analysis

For RNA sequencing analysis, two groups of 4T1 cells (3 samples per group) were first treated with PBS or Tet overnight. Total RNA was then collected using TRIzol reagent. Differential gene expression analyses were performed at Shanghai Genechem Co., Ltd.

### Animals and Tumor Model

Female BALB/c mice of 4–6 weeks of age were purchased from Nanjing University of Traditional Chinese Medicine Void Laboratory Animal Center and were performed in strict accordance with the protocol approved by the Laboratory Animal Center (Experimental Animal Use Permit: SYXK (Su) 2023‐0077; Office of Laboratory Animal Welfare Certification: F20‐00470). To prepare orthotopic murine model of breast cancer, 4T1 cells (3×10^6^) were inoculated into the breast pad of each mouse. The mice were randomly divided into 7 groups when the tumor size of about 100 mm^3^.

### Antitumor Efficacy

Each group of the tumor‐bearing mice was injected via tail vein with 100 µL of PBS, Ce6, Tet, MT@C, MC@C, MCA@C, MTCA@C NPs (at the equivalent of 2.5 mg kg^−1^ Ce6 and 15 mg kg^−1^ Tet) every twice day for a total of five times. Laser groups were subjected to 660 nm laser irradiation at a power density of 0.7 W cm^−2^ for 4 min 4 h postinjection. The mice were sacrificed at the end of the experiment and the tumors were collected for histological analysis.

### Statistical Analysis

All experiments were performed with at least three independent replicates. Data are presented as means ± SD, as indicated in the figure legends. Data collection and analysis were performed using GraphPad Prism 9.0.0 software and image J 2.0.0 software. Significant differences were evaluated using two‐tailed Student's *t*‐tests. *P* value of less than 0.05 was considered statistically significant (*p < 0.05, ** *p* < 0.01, *** *p* < 0.001).

## Conflict of Interest

The authors declare no conflict of interest.

## Supporting information



Supporting Information

## Data Availability

The data that support the findings of this study are available from the corresponding author upon reasonable request.
