# Supplementary material for: Smart Nanoparticles Disrupting Energy Supply through Triple Mechanisms to Kill Tumors via Dual Disruption of Mitochondria and Lysosomes
Source: Adv Sci (Weinh). 2025 Nov 26;13(7):e17373. doi: 10.1002/advs.202517373 (PMC12866711; doi:10.1002/advs.202517373)
Supplement: Supplementary file 1 — Supporting Information [file ADVS-13-e17373-s001.docx]

Supporting Information

Smart Nanoparticles Disrupting Energy Supply through Triple Mechanisms to Kill Tumors via Dual Disruption of Mitochondria and Lysosomes

*Xiao Xu^a,1^, Qiqing Huang^a,1^, Yang Liu^b,1^, Jinzhuo Liu^a^, Deyi Yang^c^, Yanni Song^c,*^, Xin Han^a,*^*

1. Xiao Xu, Qiqing Huang, Jinzhuo Liu, Xin Han

State Key Laboratory of Technologies for Chinese Medicine Pharmaceutical Process Control and Intelligent Manufacture, School of Medicine, Nanjing University of Chinese Medicine, Nanjing 210023, China.
E-mail: 1525@hrbmu.edu.cn; xhan0220@njucm.edu.cn

B. Yang Liu

Department of Medical Oncology, National Cancer Center/National Clinical Research Center for Cancer/Cancer Hospital & Shenzhen Hospital, Chinese Academy of Medical Sciences and Peking Union Medical College, Shenzhen 518116, China.

1. Deyi Yang, Yanni Song

Department of Breast Surgery, Harbin Medical University Cancer Hospital, 150 Haping Road, Harbin 150081, China

^1^These authors contributed equally.


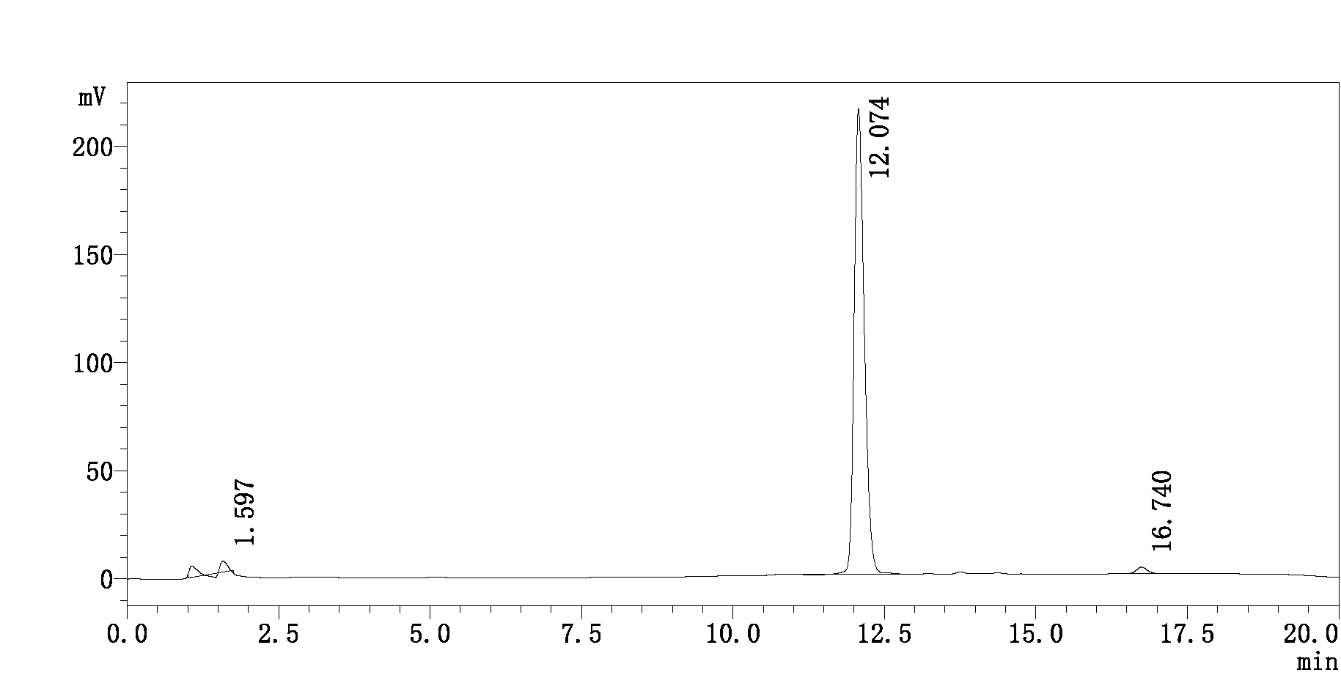


**Figure S1. HPLC analysis of Ce6-Apt (UV: 254 nm).**


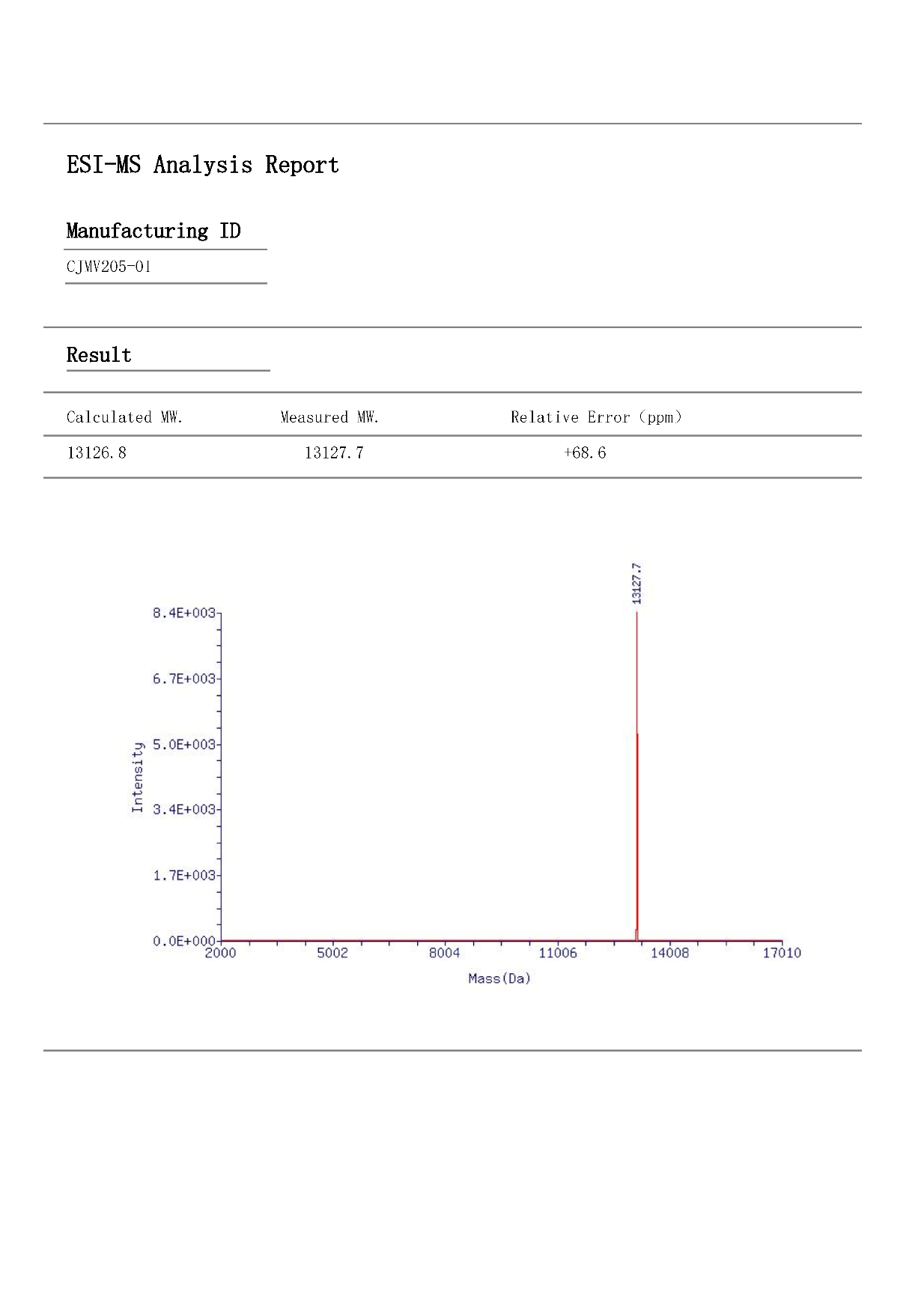


**Figure S2. ESI-MS analysis of Ce6-Apt.**

**Figure S3. XPS spectrum of H-MnO_2_ nanoparticles.**

**Figure S4. Quantification of Tet loadings at different MnO_2_ mass ratio.**


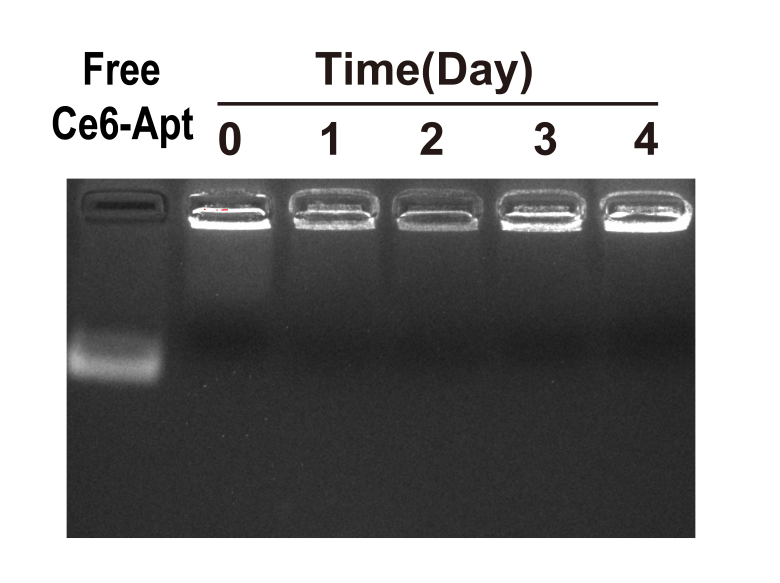


**Figure S5. Agarose gel electrophoresis analysis of Ce6-Apt release from MTCA@C NPs across five batches stored for varying periods. (pH=7.4)**

**Figure S6. Quantitative analyses of the JC-1 aggregates and JC-1 monomers fluorescence intensity.** (I: PBS; II: Ce6; III: MC@C; IV: MCA@C; V:MTCA@C ).

All data are presented as the mean ± SD (**p* < 0.05; ***p* < 0.01; ****p* < 0.001; two-tailed Student’s *t*-tests).


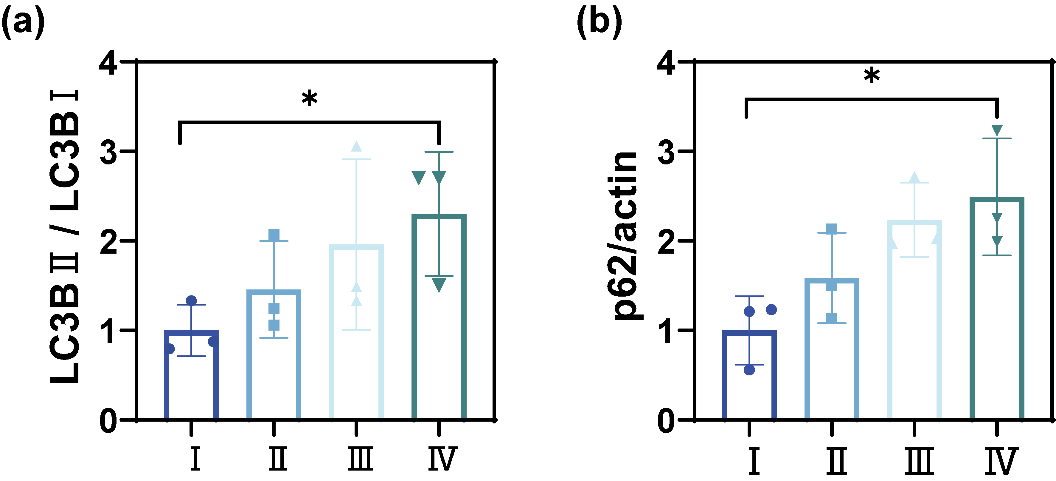


**Figure S7. Western-blot analysis of (a)** Grayscale analysis of LC3B Ⅱ/LC3B Ⅰ. **(b)** Grayscale analysis of p62/β-actin. (I: PBS; II: MC@C; III: MCA@C; IV: MTCA@C). All data are presented as the mean ± SD (**p*< 0.05; two-tailed Student’s *t*-tests).


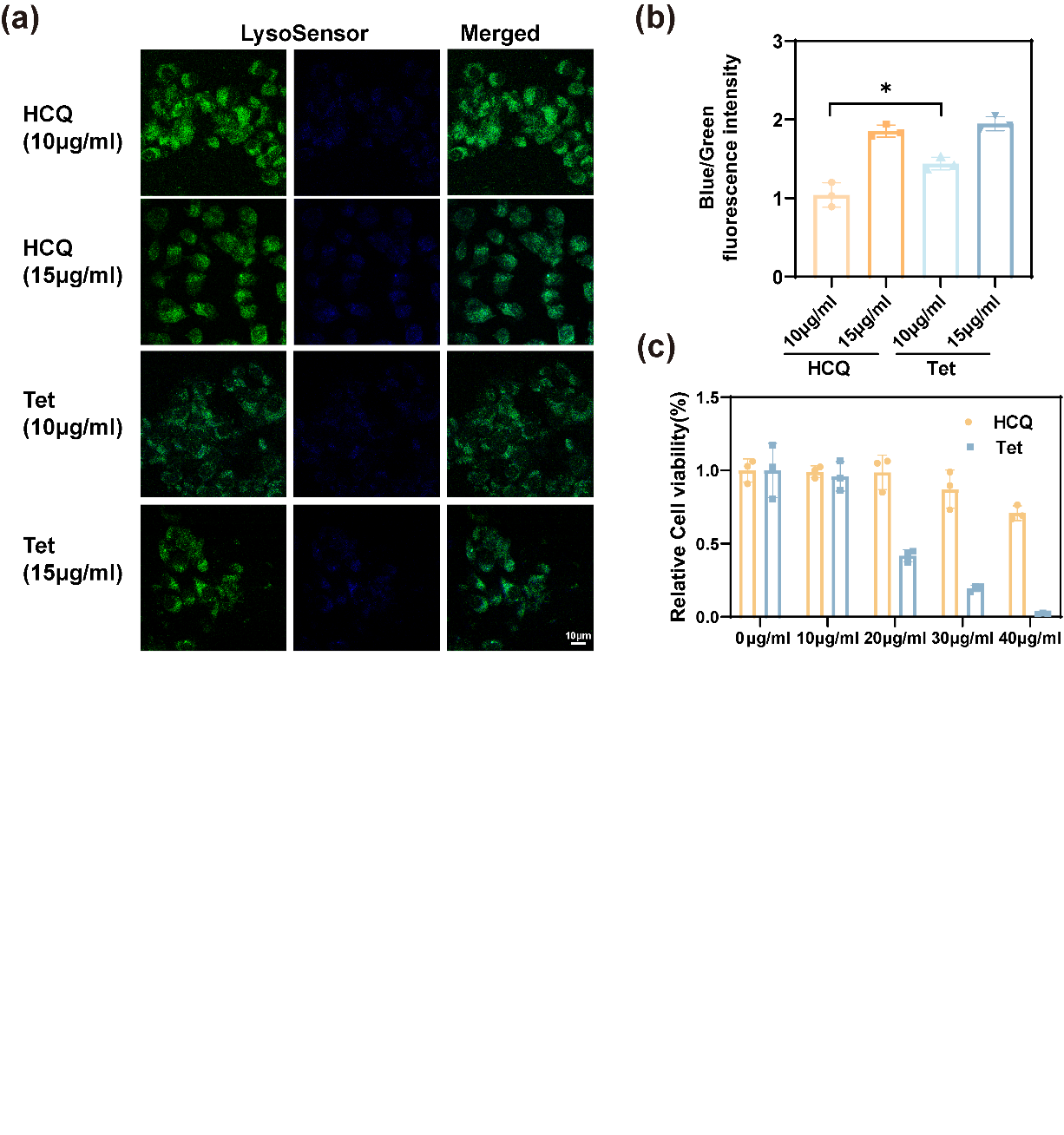


**Figure S8. Efficacy comparison of HCQ and Tet. (a)** Fluorescence of LysoSensor in 4T1 cells treated with HCQ (10 μg mL^-1^, 15 μg mL^-1^) and Tet (10 μg mL^-1^, 15 μg mL^-1^) by fluorescence microscope. (scale bar: 10 μm) **(b)** Quantitative analyses of blue and green fluorescence intensity measurements by fluorescence microscope. **(c)** CCK8 assay for 4T1 cells incubated with different concentration of HCQ or Tet for 24 hours. All data are presented as the mean ± SD (**p*< 0.05; two-tailed Student’s *t*-tests).


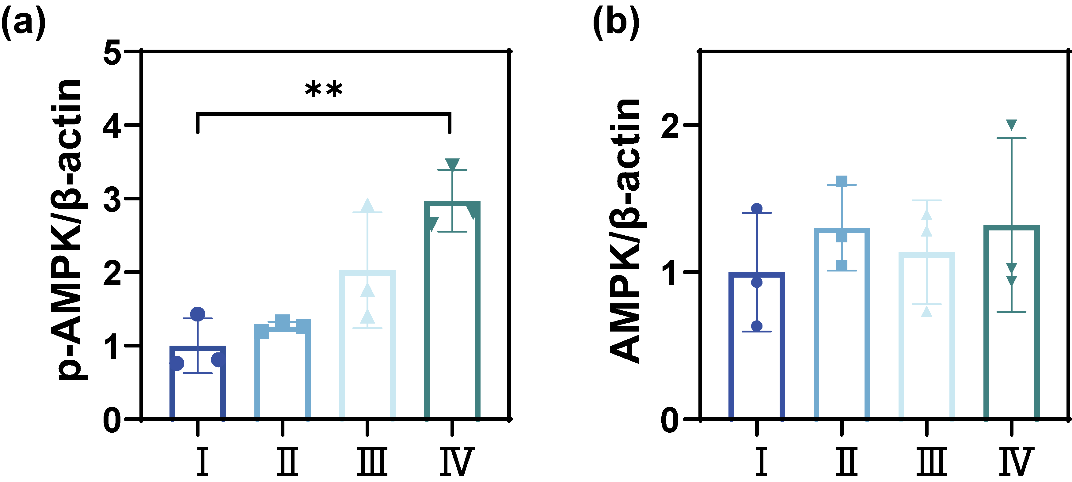


**Figure S9. Western-blot analysis of (a)** Grayscale analysis of AMPK/β-actin. **(b)** Grayscale analysis of p-AMPK/β-actin. (I: PBS, II: Tet, III: MCA@C, IV: MTCA@C). All data are presented as the mean ± SD (**p* < 0.05; ***p* < 0.01; ****p* < 0.001; two-tailed Student’s *t*-tests).


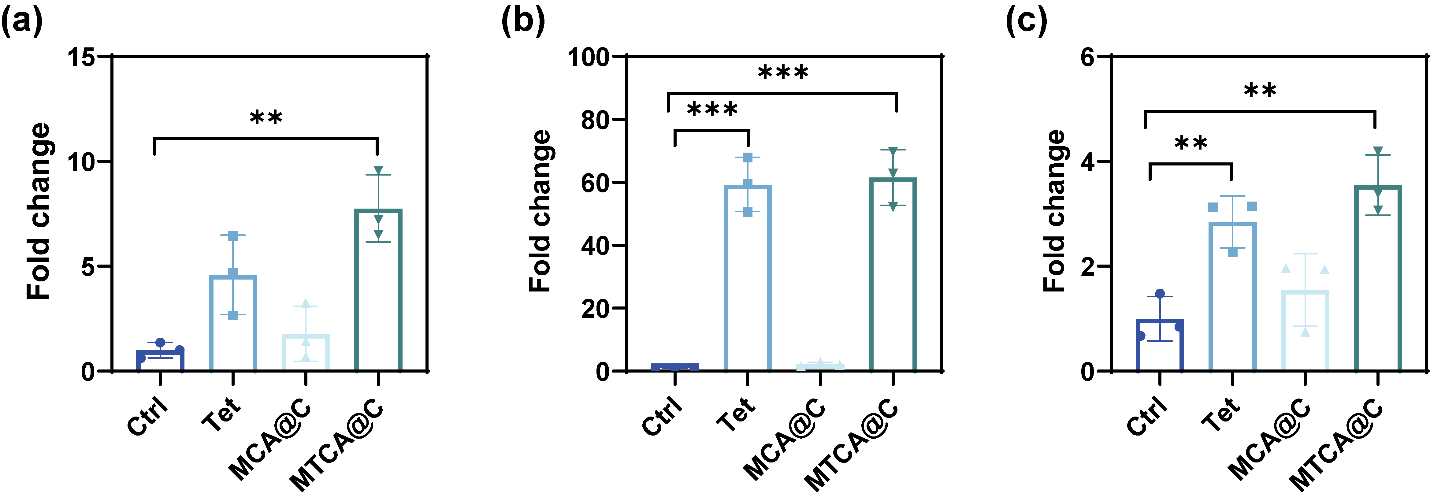


**Figure S10. Quantitative analyses of (a)** Lucifer Yellow fluorescence intensity. **(b)** Rab7 fluorescence intensity. **(c)** LAMP1 fluorescence intensity. All data are presented as the mean ± SD ( **p* < 0.05; ***p* < 0.01; ****p* < 0.001; two-tailed Student’s *t*-tests).


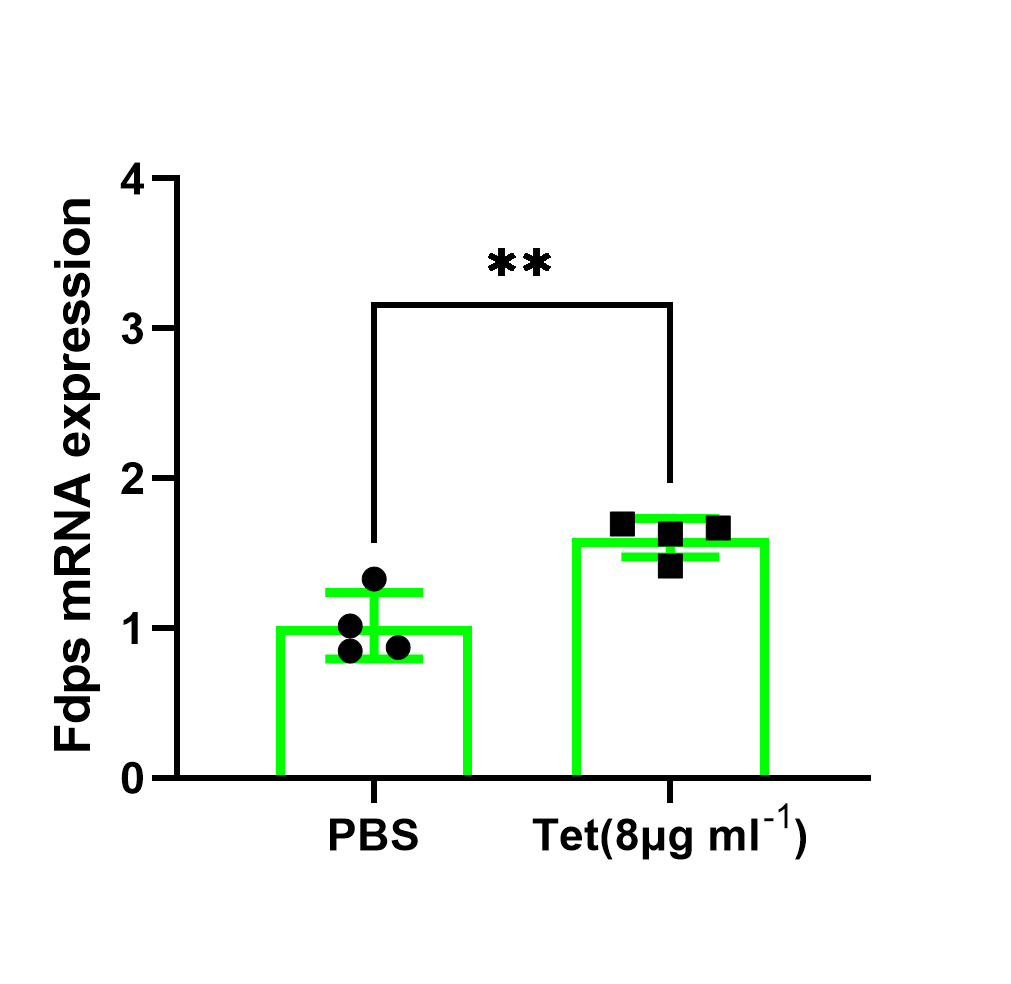


**Figure S11. Expression analysis by qRT-PCR of Fdps mRNA in 4T1 cells.** All data are presented as the mean ± SD (n=4, **p* < 0.05; ***p* < 0.01; ****p* < 0.001; two-tailed Student’s *t*-tests).


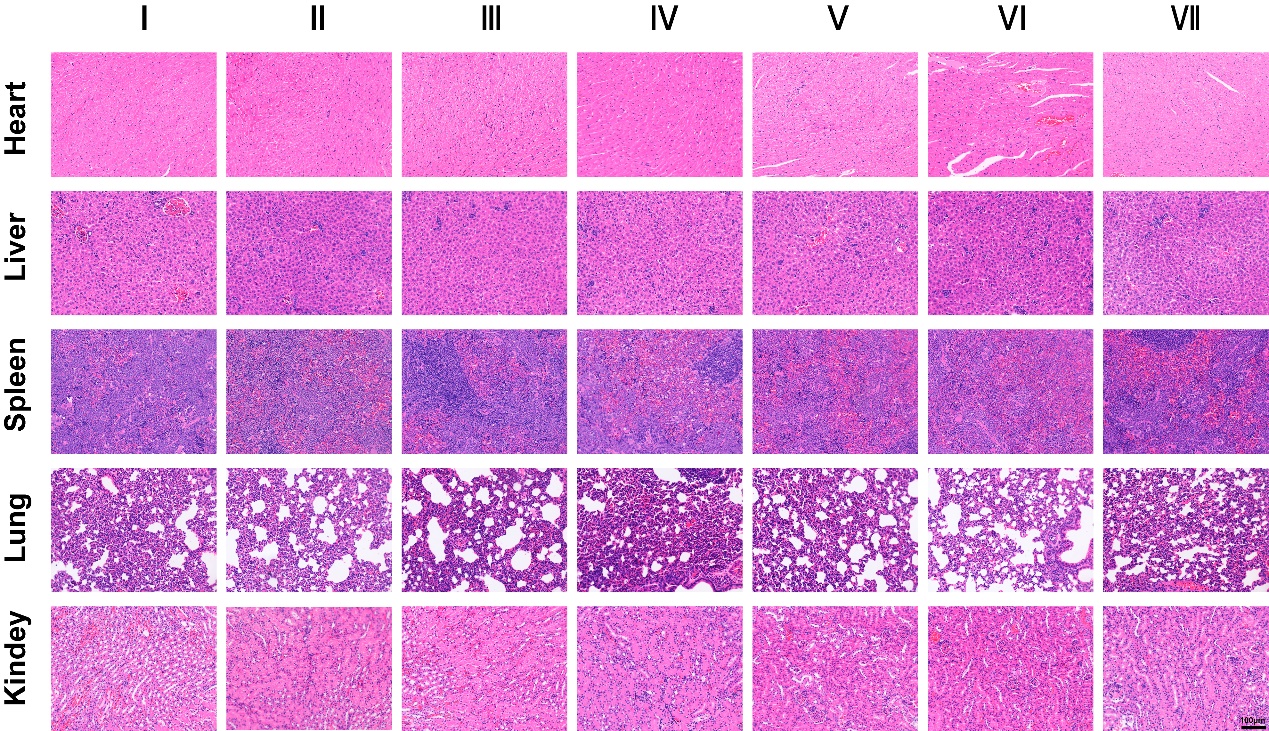


**Figure S12. H&E staining of major organs after treated with different formulations.** (I: PBS; II: Ce6; III: Tet; IV: MT@C; V: MC@C; VI: MCA@C; VII: MTCA@C) (scale bar: 100 μm).


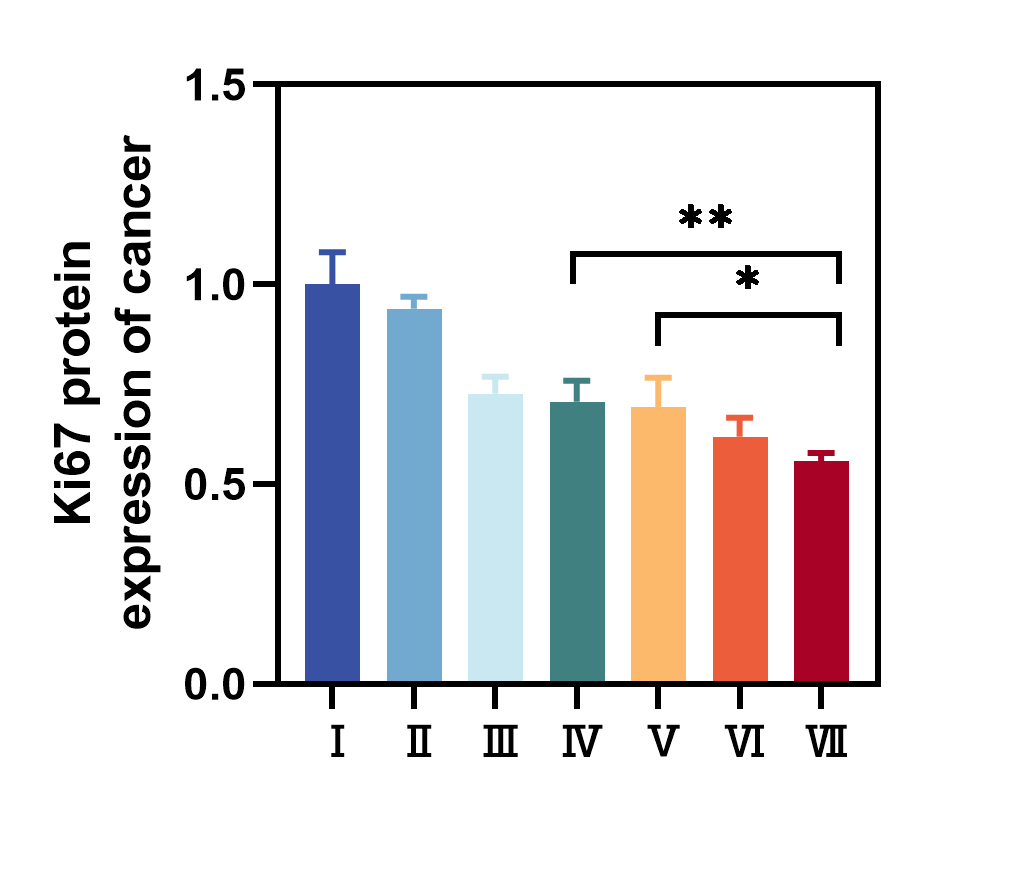


**Figure S13. Quantitative analyses of Ki67 protein expression of cancer.** (I: PBS; II: Ce6; III: Tet; IV: MT@C; V: MC@C; VI: MCA@C; VII: MTCA@C). All data are presented as the mean ± SD (n=3, **p* < 0.05; ***p* < 0.01; ****p* < 0.001; two-tailed Student’s *t*-tests).


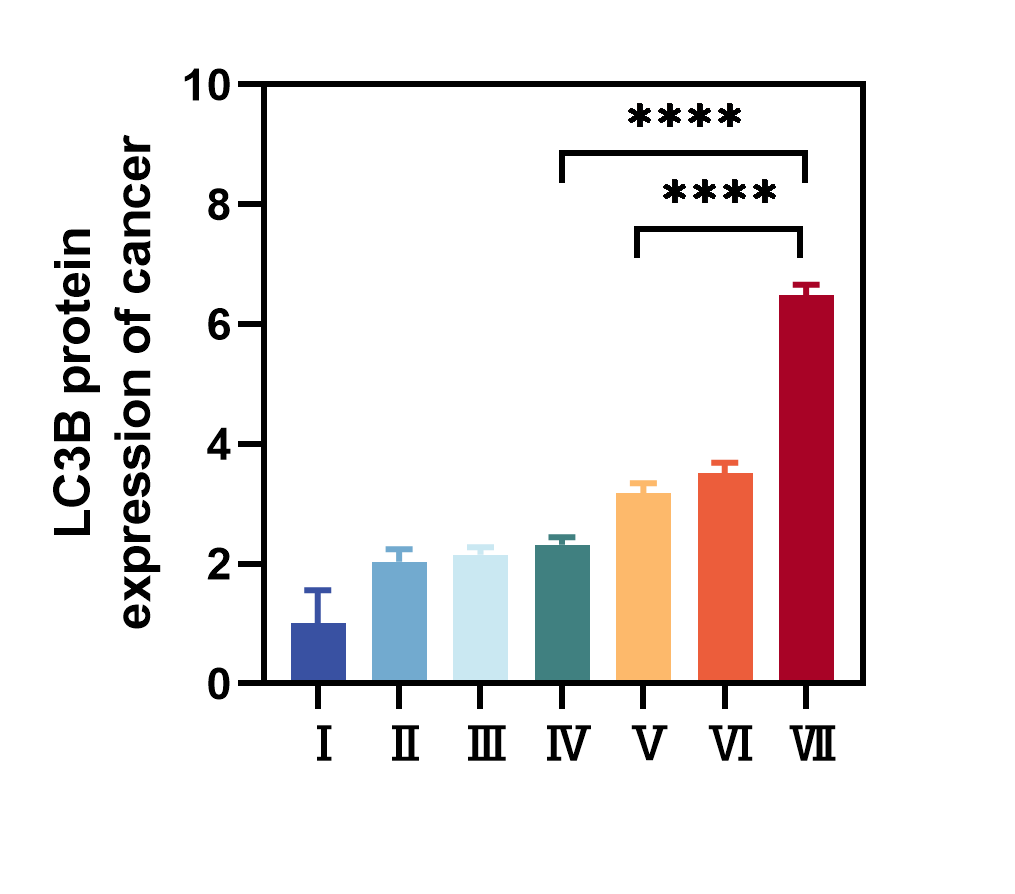


**Figure S14. Quantitative analyses of LC3B protein expression of cancer by immunohistochemistry.** (I: PBS; II: Ce6; III: Tet; IV: MT@C; V: MC@C; VI: MCA@C; VII: MTCA@C). All data are presented as the mean ± SD (n=3, **p* < 0.05; ***p* < 0.01; ****p* < 0.001; two-tailed Student’s *t*-tests).

**
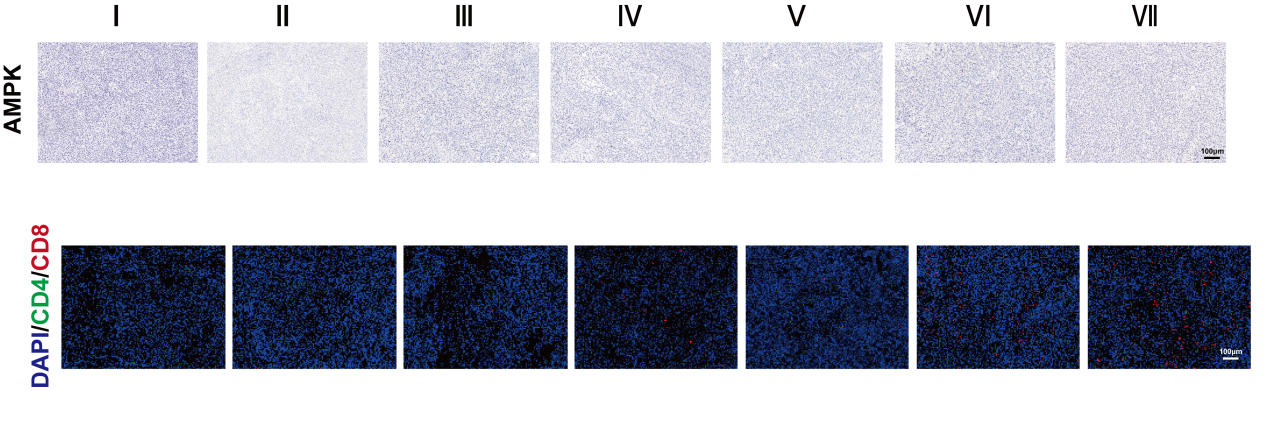
**

**Figure S15. Representative IHC analysis of AMPK in tumor tissues.** (I: PBS; II: Ce6; III: Tet; IV: MT@C; V: MC@C; VI: MCA@C; VII: MTCA@C) (scale bar: 100 μm).


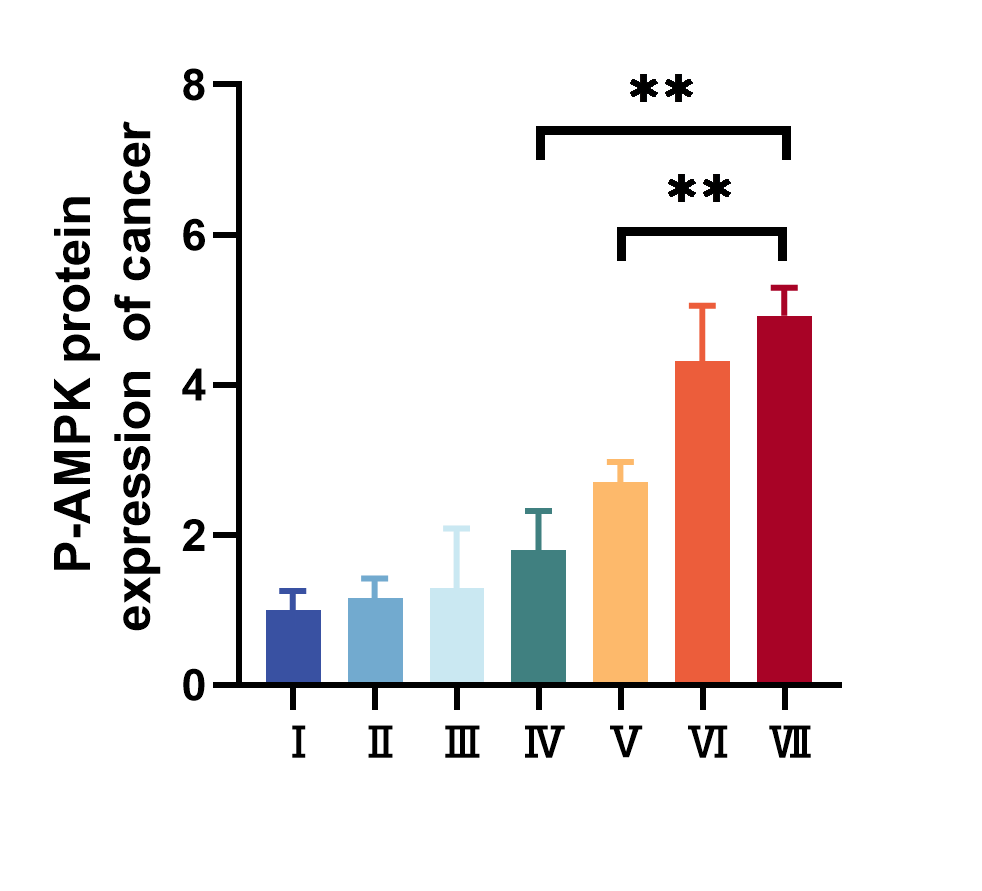


**Figure S16. Quantitative analyses of p-AMPK protein expression of cancer by immunohistochemistry.** (I: PBS; II: Ce6; III: Tet; IV: MT@C; V: MC@C; VI: MCA@C; VII: MTCA@C). All data are presented as the mean ± SD (n=3, **p* < 0.05; ***p* < 0.01; ****p* < 0.001; two-tailed Student’s *t*-tests).


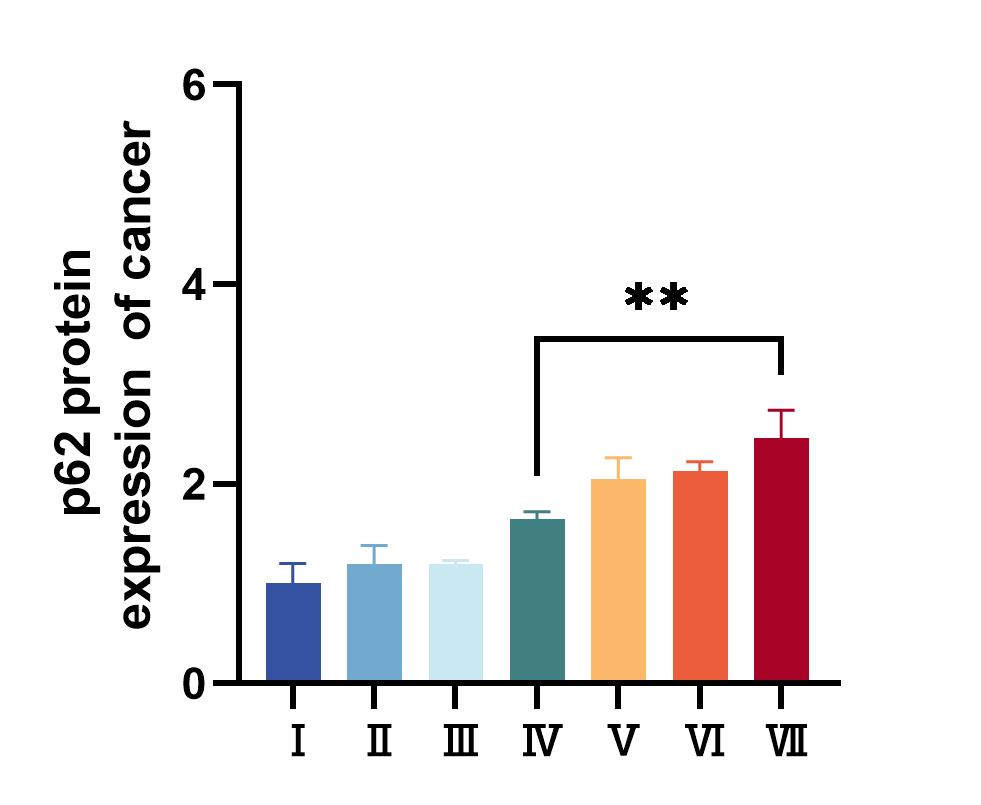


**Figure S17. Quantitative analyses of p62 protein expression of cancer by immunohistochemistry.** (I: PBS; II: Ce6; III: Tet; IV: MT@C; V: MC@C; VI: MCA@C; VII: MTCA@C). All data are presented as the mean ± SD (n=3, **p* < 0.05; ***p* < 0.01; ****p* < 0.001; two-tailed Student’s *t*-tests).


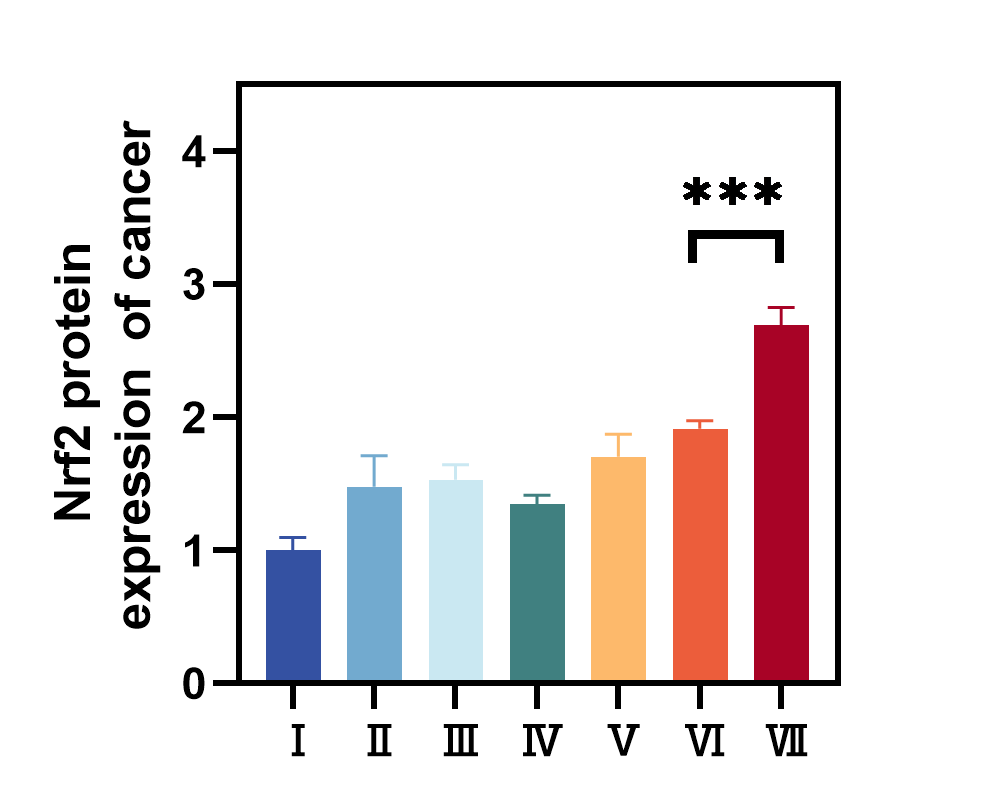


**Figure S18. Quantitative analyses of Nrf2 protein expression of cancer by immunohistochemistry.** (I: PBS; II: Ce6; III: Tet; IV: MT@C; V: MC@C; VI: MCA@C; VII: MTCA@C). All data are presented as the mean ± SD (n=3, **p* < 0.05; ***p* < 0.01; ****p* < 0.001; two-tailed Student’s *t*-tests).


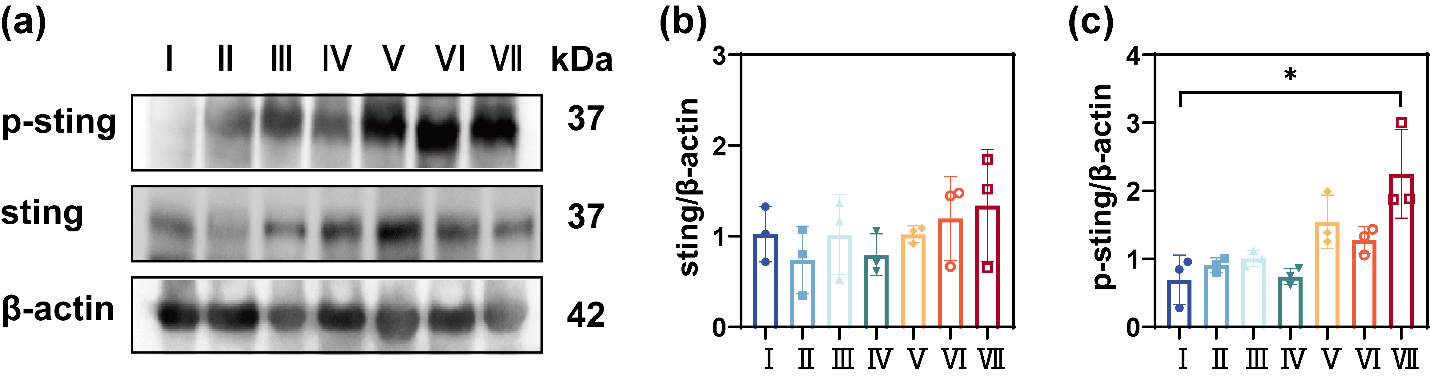


**Figure S19. Western-blot analysis (a)** protein expression of sting and p-sting in tumor tissues. β-actin was used as a control. **(b)** quantitative analyses of sting protein expression of cancer. **(c)** quantitative analyses of p-sting protein expression of cancer. (I: PBS; II: Ce6; III: Tet; IV: MT@C; V: MC@C; VI: MCA@C; VII: MTCA@C). All data are presented as the mean ± SD (n=3, **p* < 0.05; ***p* < 0.01; ****p* < 0.001; two-tailed Student’s *t*-tests).


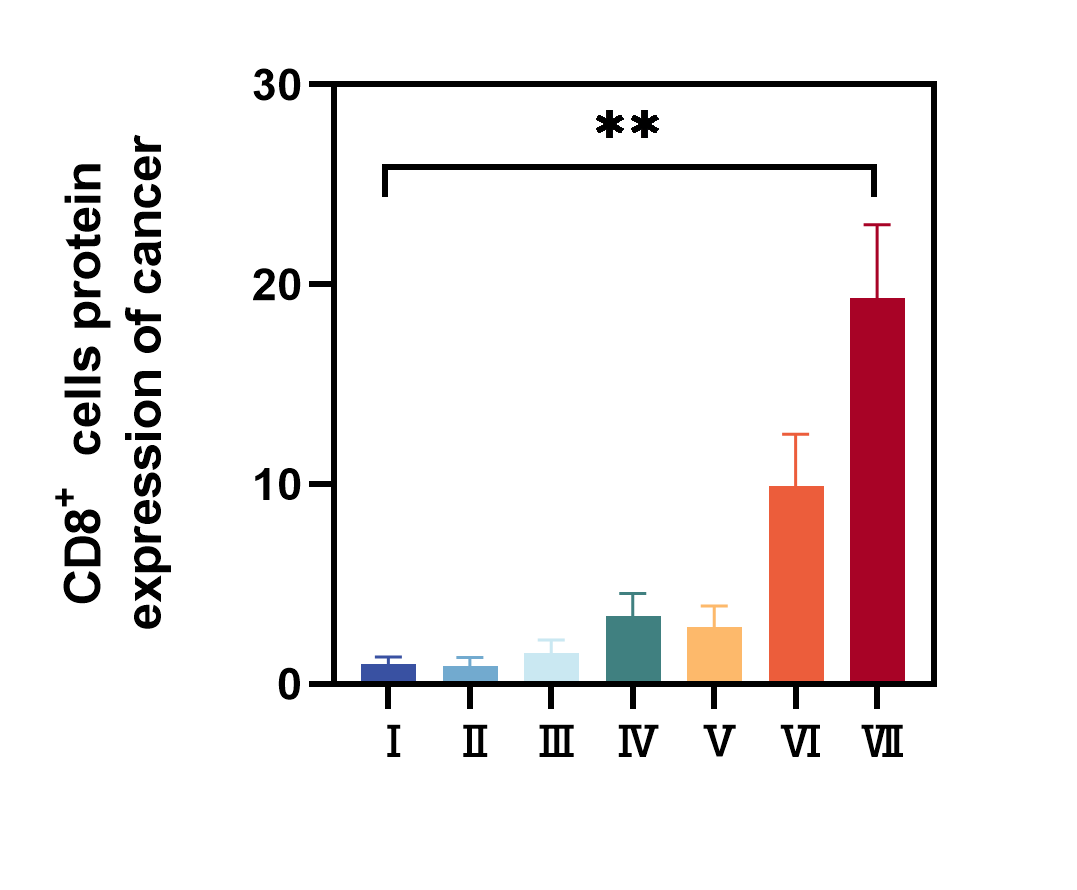


**Figure S20. Quantitative analyses of T cells in the tumor.** (I: PBS; II: Ce6; III: Tet; IV: MT@C; V: MC@C; VI: MCA@C; VII: MTCA@C). All data are presented as the mean ± SD (n=3, **p* < 0.05; ***p* < 0.01; ****p* < 0.001; two-tailed Student’s *t*-tests).

Table S1. The nucleotide sequence required for the experiment

| **Name** | **Nucleotide Sequence (5’-3’)** |
| --- | --- |
| Aptamer | CCGTGTCTGGGGCCGACCGGCGCATTGGGTACGTTGTTGC |
| Ce6-Aptamer | Ce6-CCGTGTCTGGGGCCGACCGGCGCATTGGGTACGTTGTTGC |
| FAM-Aptamer | FAM-CCGTGTCTGGGGCCGACCGGCGCATTGGGTACGTTGTTGC |

Table S2. Primers used in qRT-PCR assays.

| **Gene Name** | **Forward Primer (5’-3’)** | **Reverse Primer (5’-3’)** |
| --- | --- | --- |
| GAPDH | GTCAAGGCTGAGAACGGGAA | AAATGAGCCCCAGCCTTCTC |
| PINK1 | TGGGGAGTATGGAGCAGTCA | ATAACGAGGAACAGCGTCCG |
| Fdps | CTACAACTCCCAGAGTGCTGAG | GCACCAAGCACTCCCTGTTCT |
